# Analysis of Gene Expression Data from Non-Small Cell Lung Carcinoma Cell Lines Reveals Distinct Sub-Classes from Those Identified at the Phenotype Level

**DOI:** 10.1371/journal.pone.0050253

**Published:** 2012-11-27

**Authors:** Andrew R. Dalby, Ibrahim Emam, Raimo Franke

**Affiliations:** 1 Department of Molecular Biosciences, University of Westminster, New Cavendish Street, London, United Kingdom; 2 EMBL-EBI, Wellcome Trust Genome Campus, Hinxton, United Kingdom; 3 Department of Chemical Biology, Helmholtz Centre for Infection Research, Braunschweig, Germany; H. Lee Moffitt Cancer Center & Research Institute, United States of America

## Abstract

Microarray data from cell lines of Non-Small Cell Lung Carcinoma (NSCLC) can be used to look for differences in gene expression between the cell lines derived from different tumour samples, and to investigate if these differences can be used to cluster the cell lines into distinct groups. Dividing the cell lines into classes can help to improve diagnosis and the development of screens for new drug candidates. The micro-array data is first subjected to quality control analysis and then subsequently normalised using three alternate methods to reduce the chances of differences being artefacts resulting from the normalisation process. The final clustering into sub-classes was carried out in a conservative manner such that sub-classes were consistent across all three normalisation methods. If there is structure in the cell line population it was expected that this would agree with histological classifications, but this was not found to be the case. To check the biological consistency of the sub-classes the set of most strongly differentially expressed genes was be identified for each pair of clusters to check if the genes that most strongly define sub-classes have biological functions consistent with NSCLC.

## Introduction

The use of cell lines in biology as a replacement for whole animal studies plays a significant role in reducing the number of animal experiments that have to be carried out in bio-medical research. An important assumption is that gene expression in the cell lines reflects the expression patterns of the tissue from which it was isolated. Recently there has been growing criticism of the use of cell lines in cancer research because of problems with stability, misidentification and contamination [1], [2]. With newly developed cell-lines there is an increasing need to show how these relate to the corresponding tissues to demonstrate that these will be an effective model for studying cancer and developing new therapies [3].

The problems with using cell-lines for cancer research are further exacerbated by the diversity within a supposedly single type of cancer. Two recent studies of breast cancer have shown that there is considerable heterogeneity in transcriptomes of tumour cells. A study of the expression profiles from 2000 patients with breast cancer has shown that the data can be used to first discover and then validate subgroups [4]. In that case because of the large number amount of data it was possible to use independent datasets for discovery (997 cases) and validation (995 cases). In the second smaller study 105 samples could be divided into two robust clusters based on an analysis of a 31-gene subset [5]. This smaller study is important because they characterised circulating tumour cells, at the single cell level. It is these circulating cells which have distinct expression profiles from the breast cancer cell lines that are responsible for secondary tumours and metastasis.

Gene expression data can provide a starting point for the identification of biomarkers although there are considerable challenges to finding a signal amongst noisy data. Existing methods have used microarray data and while these methods are giving way to next generation sequencing technologies, they currently still provide a cheap, accessible and relatively easy to use alternative for gene profiling at the whole organism level [6].

A survey of median survival rates for cancer by Macmillan Cancer Support showed that whilst the median cancer survival times had increased for many types of cancers there has been little improvement in the last 40 years in lung cancer survival rates [7]. This is despite lung cancer being one of the most wide-spread cancers and also the focus of considerable research. This suggests that lung cancer may exhibit the same or an even greater degree of diversity than breast cancer. A large amount of variability would help to explain why progression to metastasis occurs so often and so quickly and why existing treatments such as radio or chemotherapy have only very limited effects.

The size of the gene expression studies available for lung cancer is much smaller than that available for breast cancer. Lung cancers can be divided into two major groups, Small Cell Lung Carcinomas and Non-Small Cell Lung Carcinoma (NSCLC). NSCLC can be further classified into three subtypes: Squamous cell carcinoma, Adenocarcinoma, and large cell carcinoma [8]. The largest current publicly available dataset contains expression data for only 54 NSCLC cell lines. This data was collected by Sos and coworkers as part of a study to show that the cell lines are representative of tumour samples and to validate their use for drug discovery [9].

In this paper the transcriptomic datasets from that study are used to look for heterogeneity in gene expression that can then be used to cluster the cell-lines. In the work of Sos and co-workers they combined phenotype screening data to distinguish cell lines that used the PI3K and MAPK pathways to suggest that a broad target therapy that targets both pathways may be an effective treatment across all NSCLC variants [10]. In this case we are taking the opposite approach. By using the gene expression variation we show how an appropriate screen can be designed that will cover the full diversity of NSCLC cell lines and provide a larger number of more specific therapeutic targets. By identifying the key genes that are differentially expressed between the clusters, new genetic markers and novel therapeutic targets can be then be identified.

## Results and Discussion

### The Dataset

The data consists of single arrays collected for each of the cell lines. There are no biological or technical replicates. From a statistical perspective the lack of replicates is a concern as without replicates there is no measure of the variability because of either the experimental conditions or because of biological variation.

**Figure 1:**
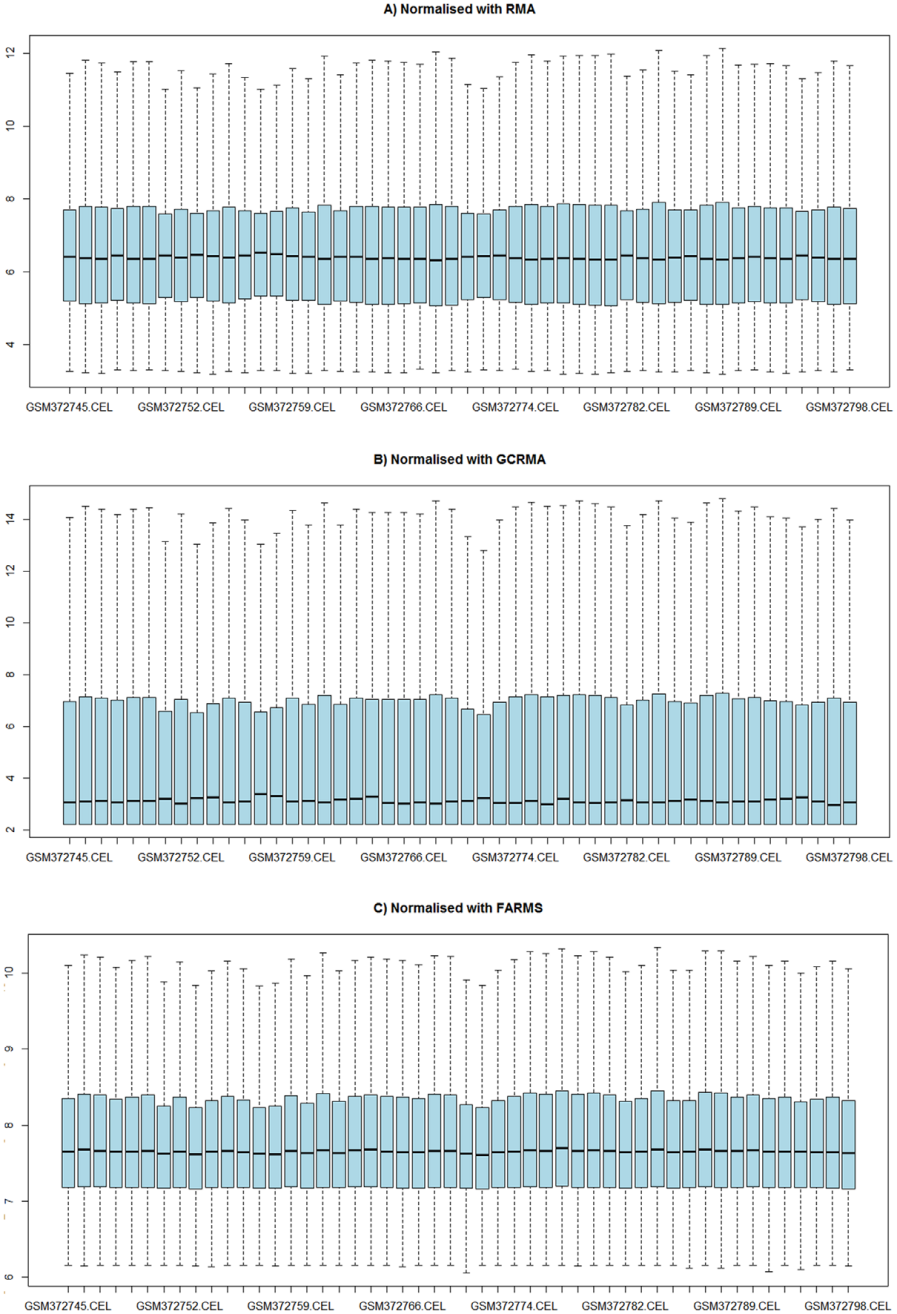
Boxplots of the log2 transformed data normalised using A) rma, B) gcrma, and C) farms.

Improvements in the production of oligonucleotide arrays have suggested that they are now highly reproducible and that there might no longer be a need for technical replicates except in the case of quality control studies [11]. The downside of carrying out more replicates is that variability can actually increase without careful design, because of the need for more sample preparations, more experimental runs that might be carried out over several days and by different experimentalists. This increases the number of variables that need to be controlled for [12]. Whether this is in fact the case or not by using strict quality control to look for anomalous effects the need for costly technical replication can be avoided or at least reduced. Quality control can be either through the design of the arrays, as in the case of adding in controls such as house-keeping genes, or through statistical methods [13].

**Table 1:** Values for the Filtering of the Microarray Probeset Level Data.

Target number or probes	Normalisation Method		Ratio (r)		Difference (d)		Actual Number of Probes	
300	rma		6.2		64		290	
	gcrma		27		64		282	
	farms		2.5		64		282	
1000	rma		4.2		64		932	
	gcrma		20		64		921	
	farms		2.0		32		957	
1A								
**Target number of probes**	**Normalisation Method**	**Lower Threshold on** **Log2 Expression**		**Multiple of** **Interquar** **tile Range**		**Median Expression**		**Actual Number of Probes**
300	rma	25% above 9		1.08		>9.3		308
	gcrma	25% above 9		1.4		>9.3		326
	farms	25% above 9		0.8		>9.3		328
1000	rma	25% above 9		0.65		>9.3		1025
	gcrma	25% above 9		0.85		>9.3		1064
	farms	25% above 9		0.5		>9.3		914
1B								

Table 1A are the parameters for the Golub filtering and Table 1B are the parameters for the median based fitting. Where **r** is the ratio between the highest and lowest level of expression for a particular probe across all the arrays and **d** is the difference between the maximum and minimum expression levels. IQR is the interquartile range and the lower threshold must be passed by at least 25% of the arrays.

Turning to the problem of a lack of biological replicates, it is very important in microarray studies to have biological replicates in order to account for the natural variation in growth and cell populations [14]. These variations can depend on a multitude of factors such as variations in the growth medium, temperature and sample handling. Unfortunately in this case there are no biological replicates the assumption was that the gene expression data comes cell lines that are assumed to be stable and biologically reproducible.

**Figure 2:**
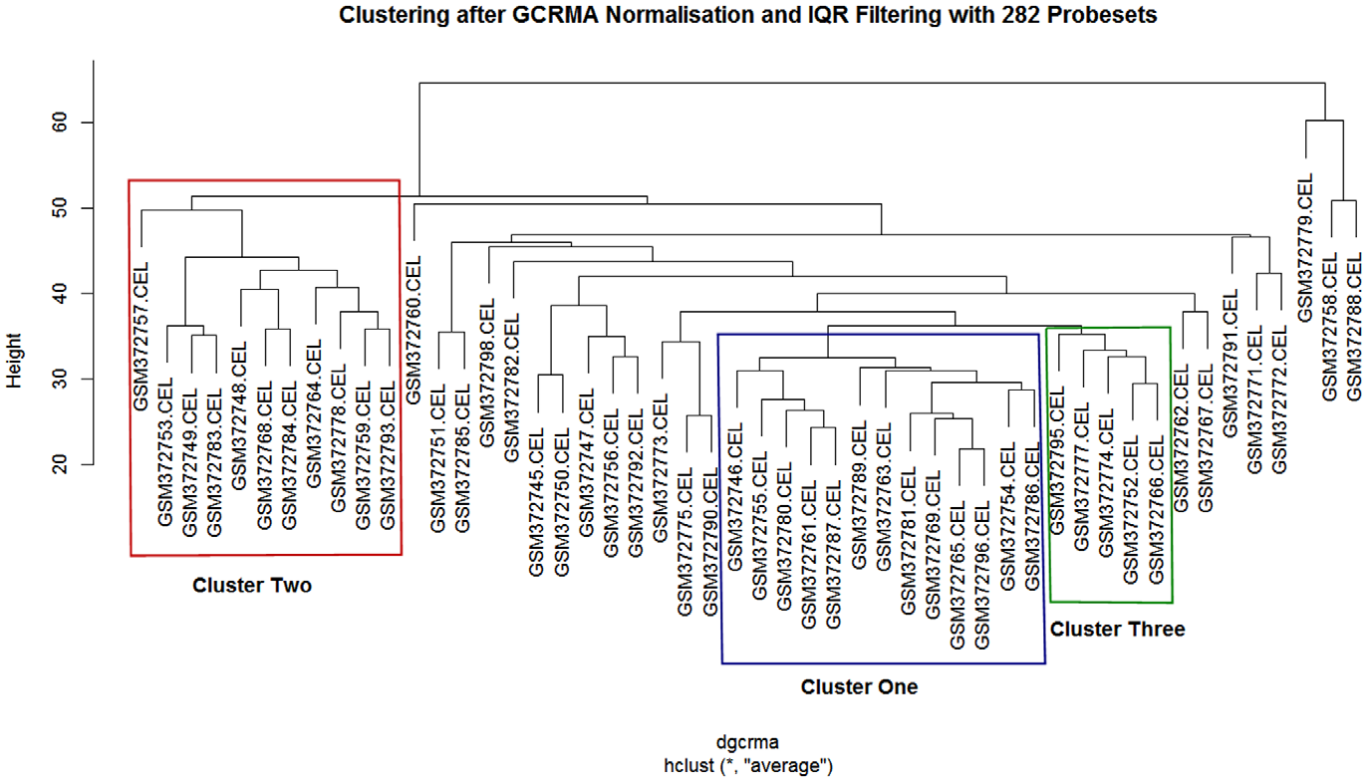
Cluster dendrogram from hierarchical agglomerative clustering of the gcrma normalised data, filtered with interquartile range filtering to give 282 probesets.

**Table 2:** Cluster Assignments and the Consensus From Analysis of the Dendrograms Produced by Agglomerative Hierarchical Clustersing of the Normalised and Filtered Datasets.

Filtering Method		Golub						Interquartile Range					
Normalisation		Farms		RMA		GCRMA		Farms		RMA		GCRMA	
No. of Probesets		L	S	L	S	L	S	L	S	L	S	L	S
Array	Consensus												
GSM372745	0				2	2		2		3	3	3	3
GSM372746	0						2	2		1	2	1	1
GSM372747	0		2					1		3	3	3	3
GSM372748	2	2	2	2	2	2	2	1	1	1	1	1	1
GSM372749	2	2	2	2	2	2	2	1	1	1	1	1	1
GSM372750	0				2	2		2	2	2	2	2	2
GSM372751	0		3					2	2	2	2	2	2
GSM372752	3	3	3	3	3	3	3	2	2	2		2	2
GSM372753	2	2	2	2	2	2	2	1				3	
GSM372754	1	1		3		1	3	1	3		3		3
GSM372755	1	1		1		1	1	1	1		1	1	1
GSM372756	3	1		3	3	3	3	1				3	3
GSM372757	0	2	2	2	2	2	2	2		3	3	3	3
GSM372758	0				2								
GSM372759	2		2		2	2	2	2	2		2	2	2
GSM372760	0	1	3	1		1	1	3	3	3	3		3
GSM372761	1	1	1	1	1	1	1	2	2	2	2	2	2
GSM372762	3	3	3	3	3	3	3	2	2	2	2	2	2
GSM372763	1	1	1	1		1	1	1	1	1	1	1	1
GSM372764	2		2		2		2			2			2
GSM372765	3	1		1		1		3	3	3	3	3	3
GSM372766	3	3		3	3	3	3	3	3	3	3		3
GSM372767	3	3	3	3	3	3	3	1	1	1	1	1	1
GSM372768	2	2	2	2	2	2	2				1	1	
GSM372769	1	1	1	1	1	1	1	3	3	3	3	3	3
GSM372771	2	2	2	2	2	2	2		1		1	1	1
GSM372772	0	2		2	2	2		2	2	2	2	2	2
GSM372773	1	1	1	1	1	1	1	2	2	2	2	2	2
GSM372774	1	1		1		1	1	1	1				1
GSM372775	1	1	1	1	1	1	1	1	1	1	1	1	1
GSM372777	1	1	1	1	1	1	1	2	2	2	2	2	2
GSM372778	0				2	2		1	3	1	1	1	1
GSM372779	0						2	1		3	3	3	3
GSM372780	1	1	1	1	1	1	1	2					
GSM372781	1			1	1	1		1	1		1	1	3
GSM372782	1	1	1	1		1	1	1	1	1	1	1	
GSM372783	2	2	2	2	2		2	1	1	1	1	1	1
GSM372784	2	2	2	2	2	2	2						
GSM372785	0		1										2
GSM372786	1	1		1	1	1	1						
GSM372787	0		2				1						
GSM372788	0							2		2	2	2	2
GSM372789	1	1	1	1		1	1	1	1	1	1	1	3
GSM372790	1	1		1	1	1	1		2	2	2	2	2
GSM372791	2	2	2	2	2	2	2	1	1	1	1	1	1
GSM372792	1	1	1	1	1	1	1	1					
GSM372793	2	2	2	2	2	2	2	1	1	1	1		
GSM372795	3	3	3	3	3	3	3						
GSM372796	1		1	1			1	2	2				
GSM372798	0							1	1	1	1	1	1

The two filtering methods are that according to Golub or using the Interquartile Range [21]. The normalisation methods are farms, rma or gcrma. The probeset sizes are approximately 1000 (L) or approximately 300 (S). The arrays are assigned to clusters 1,2,3 or 0 means there is no consensus and gaps indicate not cluster was assigned from that dendrogram.

A lack of biological replicates is far from ideal and without them we do not have a reliable measure of gene expression variance at the gene level for a single cell line. There may be biological variation between individual cells because of the stages in their life cycle this variation will be lost in the experimental samples which pool expression from a population of cells. Pooling of cell line samples has been shown to reduce the number of differentially observed genes between treated and untreated cancer cell lines [15]. This might also be the case here where differences in gene expression between cell lines will be expected to be small. The result will be fewer differentially expressed genes are observed than actually occur and a significant number of false negatives. The study will be less sensitive than it would have been had biological replicates been available but for identifying subclasses this loss of sensitivity is a less serious an issue as a large number of false positives (Type I errors).

**Table 3:** The three identified clusters and their annotations.

	Array	Cell Line	Type
**Cluster One**	GSM372754	H1648	Adenocarcinoma IIIA
	GSM372755	H1650	Adenocarcinoma IIIB
	GSM372761	H1975	Adenocarcinoma
	GSM372763	H2009	Adenocarcinoma IV
	GSM372769	H2347	Adenocarcinoma I
	GSM372773	H3122	Adenocarcinoma IV
	GSM372775	H3255	Adenocarcinoma IIIB
	GSM372777	H441	Papillary Adenocarcinoma
	GSM372780	H820	Papillary Adenocarcinoma
	GSM372781	HCC1171	Adenocarcinoma I
	GSM372782	HCC1195	Adenosquamous carcinoma I
	GSM372786	HCC193	Adenocarcinoma
	GSM372789	HCC2450	Adenosquamous carcinoma
	GSM372790	HCC2935	Adenocarcinoma
	GSM372792	HCC4006	Adenocarcinoma
	GSM372796	HCC78	Adenocarcinoma
**Cluster Two**	GSM372748	Calu6	NSCLC
	GSM372749	H1299	Large cell carcinoma
	GSM372753	H157	Squamous cell carcinoma
	GSM372759	H1792	Adenocarcinoma IV
	GSM372764	H2052	Mesothelioma IV
	GSM372768	H23	Adenocarcinoma
	GSM372771	H2882	Squamous cell carcinoma IV
	GSM372783	HCC1359	Spindle-giant cell carcinoma
	GSM372784	HCC15	Squamous cell carcinoma
	GSM372791	HCC366	Adenosquamous carcinoma
	GSM372793	HCC44	Adenocarcinoma
**Cluster Three**	GSM372752	H1437	Adenocarcinoma I
	GSM372756	H1666	Adenocarcinoma III
	GSM372762	H1993	Adenocarcinoma IIIA
	GSM372765	H2087	Adenocarcinoma I
	GSM372766	H2122	Adenocarcinoma IV
	GSM372767	H2126	Adenocarcinoma
	GSM372795	HCC515	Adenocarcinoma

After clustering members of the same sub-class are assumed to have very similar gene expression profiles and so they then provide pseudo-biological replicates for any subsequent analysis of differential gene expression. In this study this last step is carried out to check for the biological consistency of the results and not in order to identify markers for classification as the study size is too small to allow reliable prediction of markers.

### Quality Control and Normalisation

An important step in quality control of the raw array data that is often ignored is the visual inspection of the raw array images. If there is contamination this can either result in dark areas where probes are obscured or bright areas where there is excessive signal. Both of these factors will affect the results and also impair normalisation. In this case visual inspection showed that dust contamination was present on one of the arrays, GSM372797.CEL (supplementary figure 1). This creates unreliable readings over a number of probes and so the array has to be excluded.

**Figure 3:**
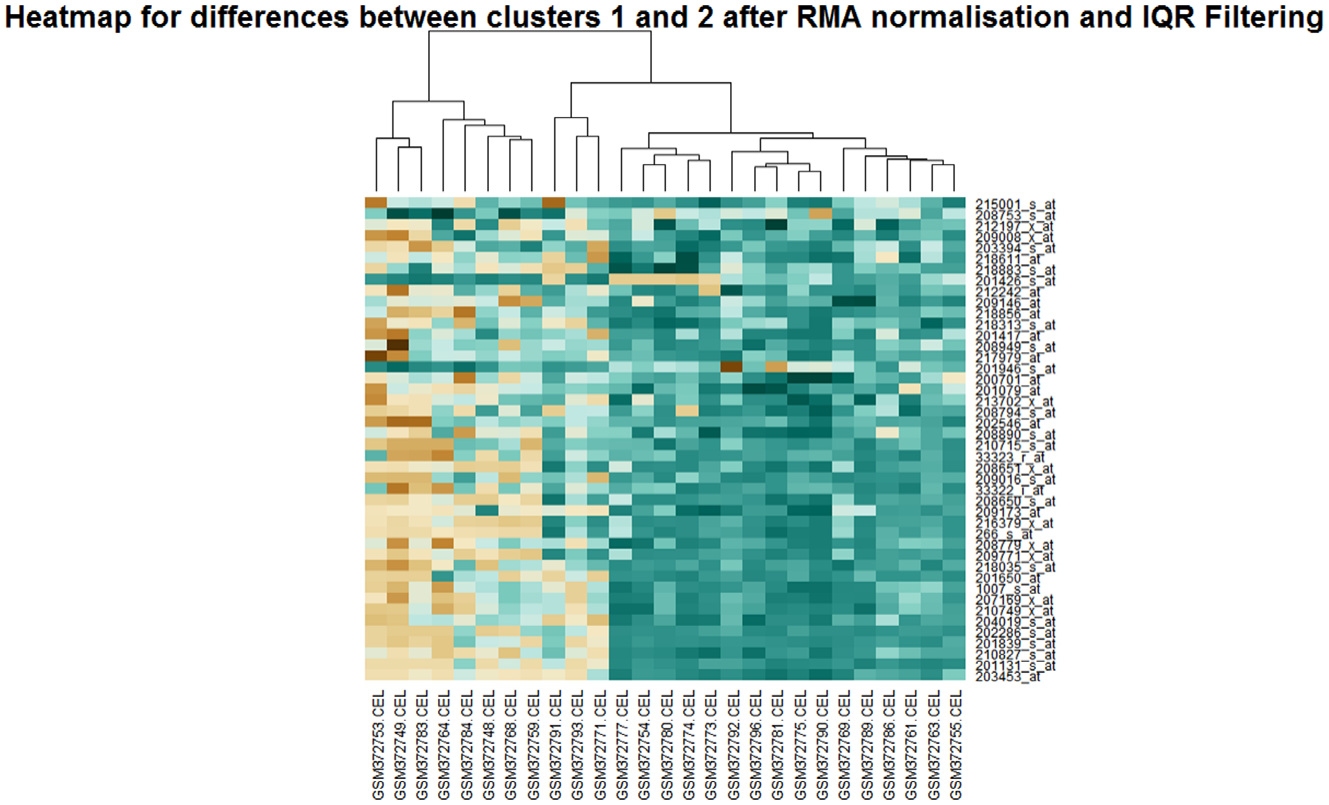
Heatmap for the differentially expressed genes between clusters 1 and 2 for the rma normalised data filtered using the IQR method to give 1025 probes.

In this case normalisation has to be carried out very carefully as the comparison of differences in gene expression is made between similar cell lines which should share very similar expression profiles. It is important to make sure that differences are genuine and not just the result of the normalisation process. Previous studies have shown that normalisation can have an effect on false discovery rate and so three normalisation methods were chosen for normalisation in this case, based on the results of the Affycomp study where normalisation methods are compared in samples where data has been spiked in [16], [17].

**Table 4:** Differentially Expressed Genes Between the Different Clusters.

Differentially Expressed Between Cluster 1 and 2	Function
SCNN1A	Sodium channel and ion regulation – signal transduction
SCEL	Sciellin – metal binding protein, epidermis development.
KRT19	Keratin 19– cytoskeletal protein.
RAB25	Member of the RAS oncogene family.
MAGE Family	Melanoma Antigen Family.
**Differentially Expressed between Cluster 1 and 3**	**Function**
TFF1	Trefoil Factor One
CPE	Carboxypeptidase E
FGG	Fibrinogen Gamma Chain - Coagulation
CPS1	Carbamoyl-phosphate Synthase – amino acid metabolism
**Differentially Expressed between Cluster 2 and 3**	**Function**
TFF1	Trefoil Factor One
FGG	Fibrinogen Gamma Chain - Coagulation
AQP3	Aquaporin 3– water reabsorption
CPE	Carboxypeptidase E
FGB	Fibrinogen Beta Chain - Coagulation
CPS1	Carbamoyl-phosphate Synthase I – amino acid metabolism

These genes are found to be differentially expressed in most of the normalisation methods and irrespective of the level of filtering.

After normalisation it is important to look at the boxplots for the distributions of expression to see if any of the arrays have outlying expression values which will affect the results of further analysis. These are likely to result from technical problems with some of the probes, this was confirmed by looking at the data for spiked in house keeping genes such as GAPDH. The unusual behaviour of the spiked in probes indicates problems with how the sample has been prepared and applied to the array. There is further evidence for this conclusion as normalisation which takes into account the probe composition (**gcrma**) eliminated the outlying expression values for these arrays [18]. This shows that there is a GC/AT bias in the sample preparation and binding. This could be a result of the amplification process or mRNA degradation. These three arrays (GSM372770.CEL, GSM372776.CEL and GSM372794.CEL) were also identified as outliers in the relative log expression for the un-normalised data and based on the consistency of these two measures they were removed from further study. It would be possible in studies where **gcrma** was the chosen method of normalisation to have included the data but there has been a study that has shown that **gcrma** has not performed as well as other methods [16].

**Figure 4:**
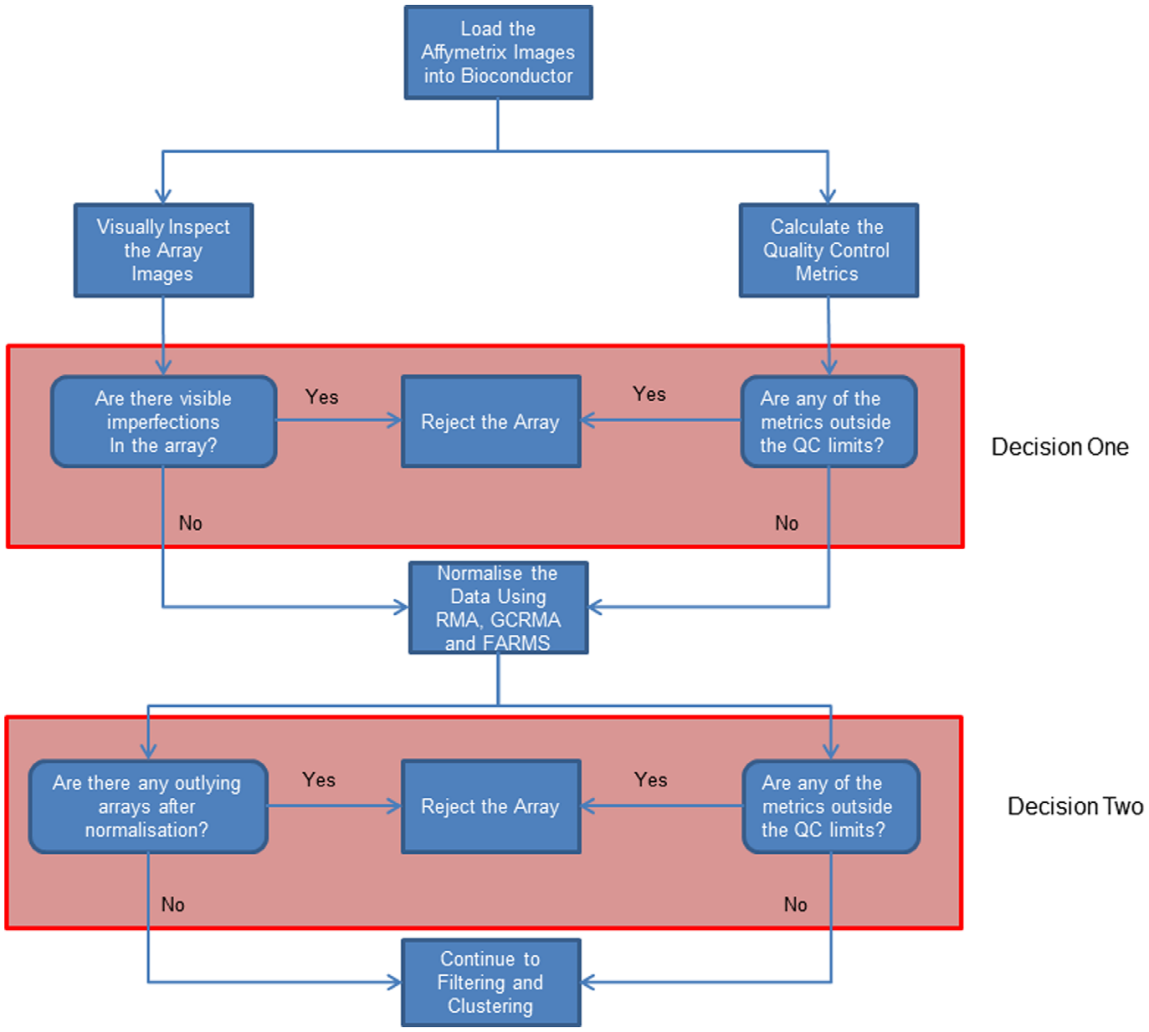
A flowchart summarising the quality control and normalisation steps of the data analysis. The pink boxes indicate when decisions are made to exclude arrays from the analysis because of quality control issues.

In this study three normalisation methods are used to reduce the chance of normalisation affecting the probesets filtered out for clustering and so these three arrays had to be excluded from further analysis. All of the normalisation methods output the expression levels on a logarithmic scale (log2) as this reduces bias from highly expressed genes. The final boxplots for the log2 expression values of the normalised data are shown in figure 1.

### Filtering

Filtering of the gene sets is necessary to deal with the statistical challenges of dealing with so many different variables and the curse of dimensionality [19]. Filtering has been shown to increase the power of subsequent statistical tests, because many fewer tests are needed [20]. With many variables it is very easy to over-fit the data to the model and even after filtering to 1000 and 300 probesets the data is still under-powered as there are only around 50 experimental data-points and ideally we need more data-points than variables for an effective model where there will not be over-fitting. Many of the genes will show negligible variation between the different cell lines and others will be strongly correlated to one another so that there will be redundancy in the data. Unfortunately without a prior knowledge of the relationships between genes we cannot do anything to reduce this redundancy as it is not clear which genes are causing the effect and which are responding to the variation in expression of this gene. In fact in a gene network cause and effect can be particularly unclear. Ideally with more data from more cell lines an iterative approach could be taken where more genes are added to the model as correlated genes are removed. In this case there is insufficient data for such a comprehensive approach. It is therefore necessary to apply a cut-off to select the most variable probesets for clustering. This is somewhat arbitrary and so filtering was carried out at two different levels to see if this affected clustering. Filtering was carried out to produce subsets of around 1000 and 300 probesets.

Golub *et al.* suggested a filtering method that depends on the ratio (r) and the difference (d) between the maximum and minimum values for the expression of a gene across all the arrays [21]. In this case it was expected that the cell lines should be very similar, especially those with the same classifications and that only a very few probesets would show any changes. The minimum difference in expression between the maximum and minimum was set to 64 which agrees with the array intensity distributions. This method was applied to the data before log transformation and so as the output of normalisation are log2 transformed measures of expression it is necessary to transform the maximum and minimum values back into the original scale to use this filtering method. There is considerable variation in the values needed to select around 1000 and 300 probes between the different normalisation methods (see table one). It is interesting that **farms** normalised data exhibits a much lower degree of variation to the other normalisations, but that **gcrma** has much lower values. The values of r and d are given in table one, along with the actual number of filtered probesets.

A more robust measure of variability is the interquartile range, as this is less sensitive to outliers with either higher or lower expression values than usual. Filtering was also carried out using this method which uses a three component filtering. The first is that over 25% of the arrays should be above the third quartile of the expression for the normalised arrays (it was set to 512 is absolute terms or 9 in log2 transformed values as the 3^rd^ quartile for the arrays was between 8 and 9), second the interquartile range should be about a threshold, third the median of the gene expression should be also be about the third quartile of the expression for the normalised arrays (in this case a cut-off of 600 in absolute units or 9.3 in log2 transformed data). The values of the interquartile ranges for this alternative filtering method and the number of probesets are also given in table one.

### Clustering

In the large-scale study for breast cancer where the researchers had 2000 cases they were able to split the data into a subset for training and a subset for validation of the clusters (subsets). In this case there are only 50 cases after normalisation, with this data the aim is to show that there are identifiable subsets within the data that have a biological explanation. As NSCLC can be divided into different sub-groups it is expected that the sub-groups should correspond to these classifications from histology. The main concern in clustering is making sure that clusters are not artefacts that arise from the normalisation process, the filtering of the genes or the clustering process itself. The simplest clustering method is agglomerative hierarchical clustering with average cluster distances. By using this method on the data from all three normalisations and the four different sets of gene filters we get twelve trees from which a consensus clustering can be calculated. By looking for clusters that are conserved in all twelve trees we can produce conservative clusters that contain a group of core members, which have very close gene expression profiles. These core clusters of cell lines can then be used to investigate which differences in gene expression distinguish the clusters. An example clustering dendrogram is given in figure 2 and a summary of the consensus clustering is given in table two. All of the cluster dendrograms are available in the supplementary material.

After consensus analysis three clusters were identified. The largest of which contains 16 cell lines, the second largest 11 cell lines and the smallest 7 cell lines. In total 35 out of 50 cell lines can be clustered into these groups. Cell-lines that could not be consistently assigned to a single group were not assigned to any cluster. For example array GSM372762 could be in either cluster 2 or 3 and so because of this ambiguity no cluster can be firmly assigned. By using twelve different normalisation and filtering combinations to generate the data for clustering and only assigning arrays that are consistently in the same cluster, this conservative approach reduces the likelihood of discovering meaningless clusters. Ideally the data should be divided into two groups, one for cluster discovery and the second for cluster validation, but in this case there is insufficient data to take this approach. Using alternative methods to test the robustness of the clusters and their sensitivity to changes in the normalisation and filtering is as much as can be done with such a small dataset.

Of the remaining cell lines that could not be clustered three are clear outliers to the rest. These are consistently at the base of the tree in all of the alternative clusterings and so are at some distance from the other cell lines. They are arrays GSM372758.CEL, GSM372779.CEL, GSM372788.CEL. These three arrays are annotated as Neuroendocrine IV, Large Cell Carcinoma, and Unknown respectively. This result suggests that these cell lines are single examples of distinctive expression types that are quite distant from the main grouping of NSCLC cell lines. These lines have been shown to be important in contributing to the gene expression signature used to distinguish lung cancer progression but as only single cell-lines for each type are currently available they are atypical of the existing NSCLC data, and so they are not ideal candidates at this time for testing novel chemical entities that are targeted across a broad spectrum of cases [22]. In the future as more examples become available these may form other distinct clusters.

The three final clusters are shown in table three along with their annotations. The largest of these contains 16 arrays. The closest of the other subsets to this group is cluster 3, while cluster 2 is a more distant group. Subset one correspond to adenocarcinoma as an annotation. This is the largest annotated group of cell lines but this has been broken down into subtypes that are not seen in the clustering. There are also a large number of adenocarcinoma cell lines that do not fall clearly into this group. The second subset has a mixture of annotations but Squamous Cell Carcinoma is the most frequent. The final cluster is too small to draw any clear conclusions about annotations but it seems to contain a mixture of histological categories. This suggests that while there is some agreement between the histological classification and the expression profile clustering, the agreement is not perfect and the expression data gives a an additional measure for classification. If we can develop a classifier based on genetic markers this will help to improve NSCLC classification methods.

### Expression Differences Between Clusters

While it is not the aim of this study to find the differentially expressed genes between the clusters identification of the most significant genes responsible for each of the clusters provides further biological support for the sub-groups.

Statistical tests can be carried out to identify the genes that are differentially expressed between the clusters. As there are a large number of tests that are carried out there has to be a correction for multiple testing and the most frequently used correction is that of Benjamini and Hochberg, as Bonferroni correction is too conservative and would mean rejecting all genes that are potentially differentially expressed [23]. With the reduced datasets of around 300 genes and 1000 genes, multiple testing is not as serious a problem as with the unfiltered dataset. Data from all three normalisations and both filtering choices were used to carry out the tests using **limma**. Figure 3 shows a heatmap of the genes responsible for distinguishing clusters one and two after **gcrma** normalisation and IQR filtering for approximately 1000 probes. Heatmaps were generated for differentially expressed genes between all of the clusters for all of the filtering and normalisation methods. While some genes are found in common between methods there is considerable variation.

The filtering method had a dramatic effect on the number of genes identified as differentially expressed between the sub-groups. In the case of clusters 1 and 3 filtered using the IQR method no genes were found to be differentially expressed at a significant level (corrected p-value of 0.01). Cluster three is a sub-group or a branching off from the main subgroup – cluster one but it was still expected that would be some significant level of differences in gene expression, but this was not the case when IQR is the filtering method. However in the case of using the Golub method for filtering, there are a number of significantly differentially expressed genes. This suggests that either the IQR filtering is losing some of the diversity in gene expression by being too stringent leading to type II errors, or that the Golub filtering method is prone to type I errors by finding signal amongst the noise. This was a consistently identified sub-group and so some feature in the gene expression variation must be responsible for distinguishing this cluster from its neighbours even if this is below the threshold of statistical significance.

Most often the 1000 gene sets gave a larger number of statistically significant differentially expressed genes between the clusters than the 300 gene sets. However the ordering of the significance of the genes was different and there was poor agreement between the gene sets identified by both the different filtering methods using the same normalisation method as well as between the normalisation methods (see supplementary data). A small number of genes were conserved as differentially expressed between the clusters in a significant number of the normalisation and filtering variants. A selection of these conserved genes are given in table four. Some are genes often labelled as oncogenes but there are others that are not usually associated. A complete list of the differentially expressed genes between the clusters for the different normalisation methods and different filtering methods as well as their annotations is given in the supplementary materials.

The final check on the validity of any microarray analysis is to see if the results make biological sense. Is differential expression of these genes between NSCLC sub-groups reasonable, and have the genes been associated with cancer in other studies? In many cases there is other prior evidence of a connection between the commonly found genes responsible for distinguishing sub-groups and other cancer studies. Of the genes in table four all but one of them has an existing link to cancer in the literature. Trefoil factor one deficiency has already been identified as causing increased tumorigenicity in human breast cancer cells [24]. Fibrinogen has been used as a factor associated with cancer mortality, but it had been assumed that this was a direct response to factors such as smoking by healthy cells, rather than any direct involvement in tumour cells [25]. Two studies have shown that Aquaporins are associated with lung cancers [26]. Carboxypeptidase E over-expression has been associated with cancer metastasis [27]. The identification of SCEL as a potential gene involved in differential expression is interesting as the gene has been associated with esophagel squamous cell carcinoma, which lends further support to cluster 2 being associated with squamous cell carcinoma [28]. Keratin 19 is already used as a biomarker for detecting circulating lung cancer cells [29]. Finally there is also tentative evidence that CPS1 might also be involved in cancer although its role is currently unclear [30].

A cause for concern is the inconsistency in genes identified as having significantly different expression levels between the three clusters using the three different normalisation methods. These discrepancies show that in this case normalisation plays a part in determining which genes are identified as significantly differentially expressed between clusters. This should not be true and hampers the reliability of gene expression analysis as well as casting doubt on genetic markers that have been identified using these techniques. Here the differences in expression levels should be small as the arrays are all for similar cell lines and it is possible that this might be an artefact of the small differences being amplified in different ways during the normalisation process, but it does raise questions about objectivity of normalisation methods, and suggests that the use of multiple normalisation methods might improve the reproducibility of gene expression analysis [16].

### Conclusion

This analysis has shown that there is heterogeneity in gene expression between the NSCLC cell lines, and that this diversity can be used to divide the cell lines into different groups that do not completely agree with the histological annotations. Lung cancer was chosen for this study because of the potential variability in its genetic make-up, which may be responsible for its rapid progression and the difficulty in developing a successful treatment. The variability between sub-classes suggests that it might not be possible to develop a broad ranging treatment, but this also presents an opportunity to develop more specific treatments and for improved molecular diagnostics. By screening new chemical entities against cell-lines chosen from all three sub-classes we can hope to generate a broad specificity drug, or alternatively the focus can be moved to targeting patients who fall into one of the subsets for a more specific treatment.

This paper has only used a small of samples where gene expression profiles were available and so it can only provide initial evidence for the existence of at least three sub-groups of NSCLC, although it is likely that with larger datasets more subgroups will be discovered. Considerable effort is required to confirm, and develop these findings in order to advance our understanding of NSCLC, to the same level as breast cancer. As larger studies containing more cell lines and gene expression data from biopsy or single cell samples become available this diversity is likely to increase further. With more biological replicates it will also be possible to identify biological markers for the sub-classes and classification methods in a robust manner. Questions have been raised about the influence of normalisation on the results of gene expression analysis but these could also be addressed by improved experimental designs that include more biological replicates and improvements in normalisation methods.

## Materials and Methods

Raw data was downloaded from ArrayExpress with accession ‘EGEOD-14925’ [31]. Quality assessment was carried out using package **arrayQualityMetrics** within Bioconductor to assess the quality of 54 CEL files included in this study [32], [33]. Two runs of quality control were carried out: before and after pre-processing. Spatial artefacts were checked for visually and array GSM372797 was found to contain contamination from dust. Therefore this array was excluded from further analysis.

Normalisation methods for Affymetrics arrays have been tested as part of Affycomp where spiked in controls are used to evaluate performance [17]. Three of the best normalisation methods are **rma**, **gcrma** and **farms** [18], [34]. The remaining arrays were normalised using the three methods within Bioconductor [32]. The resulting normalised data was again subjected to quality control analysis to look for arrays that had expression outliers in the boxplots. This showed that there were problems with three more arrays which were excluded from the rest of the study. These were arrays GSM372770, GSM372776 and GSM372794. After these arrays had been removed the data was again renormalized using the three methods. The procedure for quality control and normalisation is summarised in the flowchart in figure 4. Data from quality control metrics is combined with other measures at the decision points (shown as boxes in pink) to identify arrays where quality control indicates there are problems. These arrays are then excluded from subsequent analysis.

The data were then filtered to obtain datasets containing around 1000 and 300 genes which can then be used for cluster analysis and to determine differential gene expression. The cut-offs for filtering were different for the three normalisation methods as the normalised data has a different scale and spread from the different normalisation methods. This is why filtering cut-offs select around a target number of genes rather than an exact number. The values used for filtering using either the method from Golub *et al.* which uses differences between the maximum and minimum expression level of a gene as well as a second filtering method based on the interquartile range are given in table one. Filtering was carried out using the **genefilter** module within Bioconductor [35].

Clustering was carried out using agglomerative hierarchical clustering for both the 1000 gene and 300 gene datasets. This is perhaps the simplest possible method of clustering. There was a good degree of consistency in the clustering between the three different normalisation methods and the different filtering strategies. One cluster was particularly clearly identified in all cases. This resulted in the identification of 3 main subgroups within the arrays, that were found consistently across all three normalisation methods and the filtered datasets. Arrays that were consistently found in the same cluster regardless of normalisation method make up the core of the cluster. This resulted in 3 conservative groupings containing at least six members and containing 34 out of total of 50 arrays. These clusters were then used for differential gene expression analysis using **limma** within Bioconductor to carry out multiple testing [36]. Benjamini and Hochberg's method was used to assign a corrected p-value of 0.01 [23].

## Supporting Information

Figure S1 **An image of the raw Affymetrix array showing bright lines from dust contamination that span multiple probes.**
(TIFF)

Figure S2 **Dendrogram for FARMS normalised data using Golub filtering for 300 probes.**
(TIFF)

Figure S3 **Dendrogram for FARMS normalised data using Golub filtering for 1000 probes.**
(TIFF)

Figure S4 **Dendrogram for FARMS normalised data using IQR filtering for 300 probes.**
(TIFF)

Figure S5 **Dendrogram for FARMS normalised data using IQR filtering for 1000 probes.**
(TIFF)

Figure S6 **Dendrogram for GCRMA normalised data using Golub filtering for 300 probes.**
(TIFF)

Figure S7 **Dendrogram for GCRMA normalised data using Golub filtering for 1000 probes.**
(TIFF)

Figure S8 **Dendrogram for GCRMA normalised data using IQR filtering for 300 probes.**
(TIFF)

Figure S9 **Dendrogram for GCRMA normalised data using IQR filtering for 1000 probes.**
(TIFF)

Figure S10 **Dendrogram for RMA normalised data using Golub filtering for 300 probes.**
(TIFF)

Figure S11 **Dendrogram for RMA normalised data using Golub filtering for 1000 probes.**
(TIFF)

Figure S12 **Dendrogram for RMA normalised data using IQR filtering for 300 probes.**
(TIFF)

Figure S13 **Dendrogram for RMA normalised data using IQR filtering for 1000 probes.**
(TIFF)

Figure S14 **Heatmap for the differentially expressed genes between clusters 1 and 2 after Normalisation with FARMS and IQR filtering for 300 probes.**
(TIFF)

Figure S15 **Heatmap for the differentially expressed genes between clusters 1 and 2 after Normalisation with FARMS and IQR filtering for 1000 probes.**
(TIFF)

Figure S16 **Heatmap for the differentially expressed genes between clusters 1 and 2 after Normalisation with FARMS and Golub filtering for 300 probes.**
(TIFF)

Figure S17 **Heatmap for the differentially expressed genes between clusters 1 and 2 after Normalisation with FARMS and Golub filtering for 1000 probes.**
(TIFF)

Figure S18 **Heatmap for the differentially expressed genes between clusters 1 and 2 after Normalisation with GCRMA and IQR filtering for 300 probes.**
(TIFF)

Figure S19 **Heatmap for the differentially expressed genes between clusters 1 and 2 after Normalisation with GCRMA and IQR filtering for 1000 probes.**
(TIFF)

Figure S20 **Heatmap for the differentially expressed genes between clusters 1 and 2 after Normalisation with GCRMA and Golub filtering for 300 probes.**
(TIFF)

Figure S21 **Heatmap for the differentially expressed genes between clusters 1 and 2 after Normalisation with GCRMA and Golub filtering for 1000 probes.**
(TIFF)

Figure S22 **Heatmap for the differentially expressed genes between clusters 1 and 2 after Normalisation with RMA and IQR filtering for 300 probes.**
(TIFF)

Figure S23 **Heatmap for the differentially expressed genes between clusters 1 and 2 after Normalisation with RMA and IQR filtering for 1000 probes.**
(TIFF)

Figure S24 **Heatmap for the differentially expressed genes between clusters 1 and 2 after Normalisation with RMA and Golub filtering for 300 probes.**
(TIFF)

Figure S25 **Heatmap for the differentially expressed genes between clusters 1 and 2 after Normalisation with RMA and Golub filtering for 1000 probes.**
(TIFF)

Figure S26 **Heatmap for the differentially expressed genes between clusters 1 and 3 after Normalisation with FARMS and Golub filtering for 300 probes**
(TIFF)

Figure S27 **Heatmap for the differentially expressed genes between clusters 1 and 3 after Normalisation with FARMS and Golub filtering for 1000 probes**
(TIFF)

Figure S28 **Heatmap for the differentially expressed genes between clusters 1 and 3 after Normalisation with GCRMA and Golub filtering for 300 probes**
(TIFF)

Figure S29 **Heatmap for the differentially expressed genes between clusters 1 and 3 after Normalisation with GCRMA and Golub filtering for 1000 probes**
(TIFF)

Figure S30 **Heatmap for the differentially expressed genes between clusters 1 and 3 after Normalisation with RMA and Golub filtering for 300 probes**
(TIFF)

Figure S31 **Heatmap for the differentially expressed genes between clusters 1 and 3 after Normalisation with RMA and Golub filtering for 1000 probes**
(TIFF)

Figure S32 **Heatmap for the differentially expressed genes between clusters 2 and 3 after Normalisation with FARMS and IQR filtering for 300 probes.**
(TIFF)

Figure S33 **Heatmap for the differentially expressed genes between clusters 2 and 3 after Normalisation with FARMS and IQR filtering for 1000 probes.**
(TIFF)

Figure S34 **Heatmap for the differentially expressed genes between clusters 2 and 3 after Normalisation with FARMS and Golub filtering for 300 probes.**
(TIFF)

Figure S35 **Heatmap for the differentially expressed genes between clusters 2 and 3 after Normalisation with FARMS and Golub filtering for 1000 probes.**
(TIFF)

Figure S36 **Heatmap for the differentially expressed genes between clusters 2 and 3 after Normalisation with GCRMA and IQR filtering for 300 probes.**
(TIFF)

Figure S37 **Heatmap for the differentially expressed genes between clusters 2 and 3 after Normalisation with GCRMA and IQR filtering for 1000 probes.**
(TIFF)

Figure S38 **Heatmap for the differentially expressed genes between clusters 2 and 3 after Normalisation with GCRMA and Golub filtering for 300 probes.**
(TIFF)

Figure S39 **Heatmap for the differentially expressed genes between clusters 2 and 3 after Normalisation with GCRMA and Golub filtering for 1000 probes.**
(TIFF)

Figure S40 **Heatmap for the differentially expressed genes between clusters 2 and 3 after Normalisation with RMA and IQR filtering for 300 probes.**
(TIFF)

Figure S41 **Heatmap for the differentially expressed genes between clusters 2 and 3 after Normalisation with RMA and IQR filtering for 1000 probes.**
(TIFF)

Figure S42 **Heatmap for the differentially expressed genes between clusters 2 and 3 after Normalisation with RMA and Golub filtering for 300 probes.**
(TIFF)

Figure S43 **Heatmap for the differentially expressed genes between clusters 2 and 3 after Normalisation with RMA and Golub filtering for 1000 probes.**
(TIFF)

Table S1 **This contains the annotations that were added to the normalised data in Bioconductor including the clusters.**
(TXT)

Table S2 **This contains the full list of differentially expressed genes between all of the clusters.**
(TXT)
