# Supplementary figures and images for: Analysis of Gene Expression Data from Non-Small Cell Lung Carcinoma Cell Lines Reveals Distinct Sub-Classes from Those Identified at the Phenotype Level
Source: PLoS One. 2012 Nov 27;7(11):e50253. doi: 10.1371/journal.pone.0050253 (PMC3507731; doi:10.1371/journal.pone.0050253)

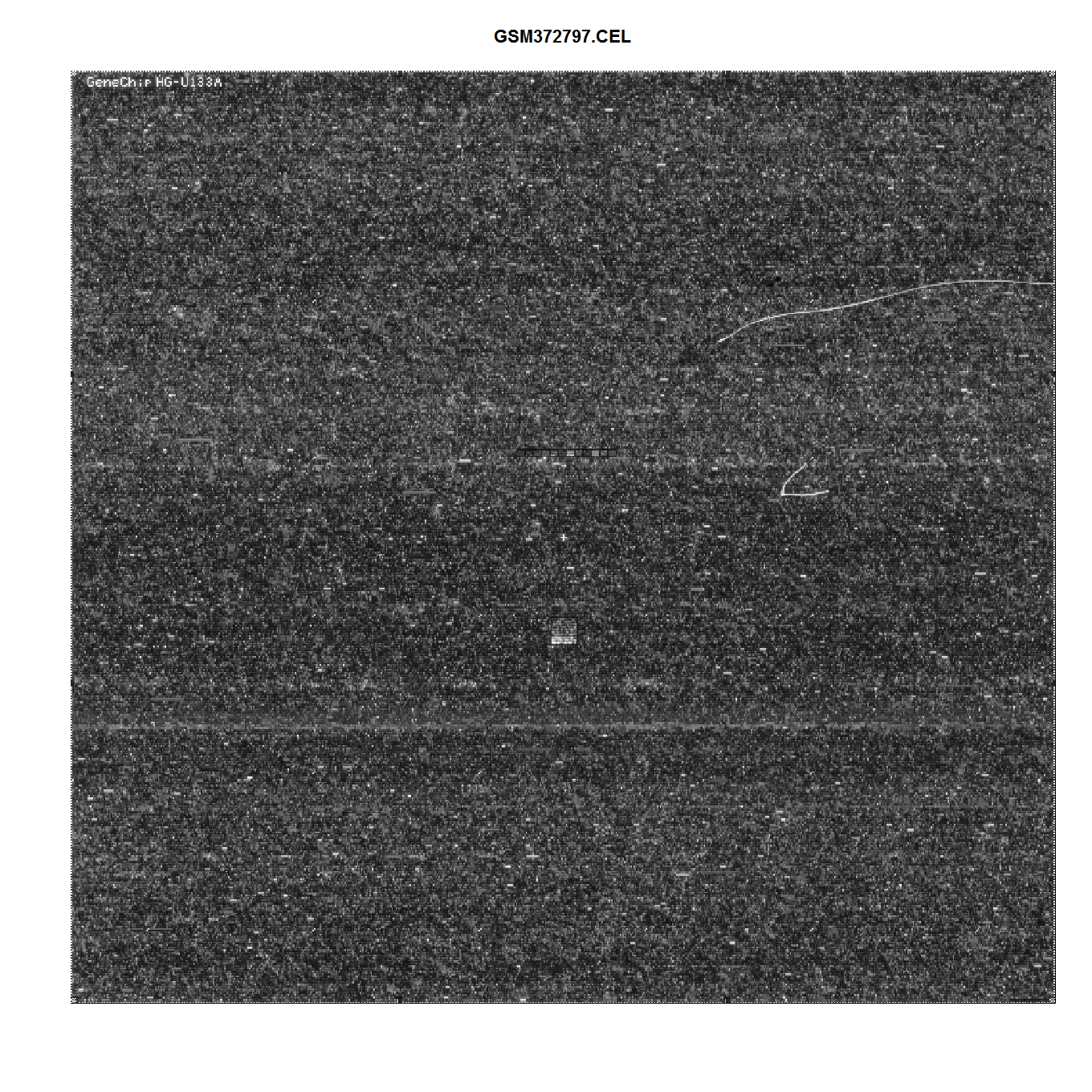

Supplement: Figure S1 — An image of the raw Affymetrix array showing bright lines from dust contamination that span multiple probes. (TIFF) [file pone.0050253.s001.tif]

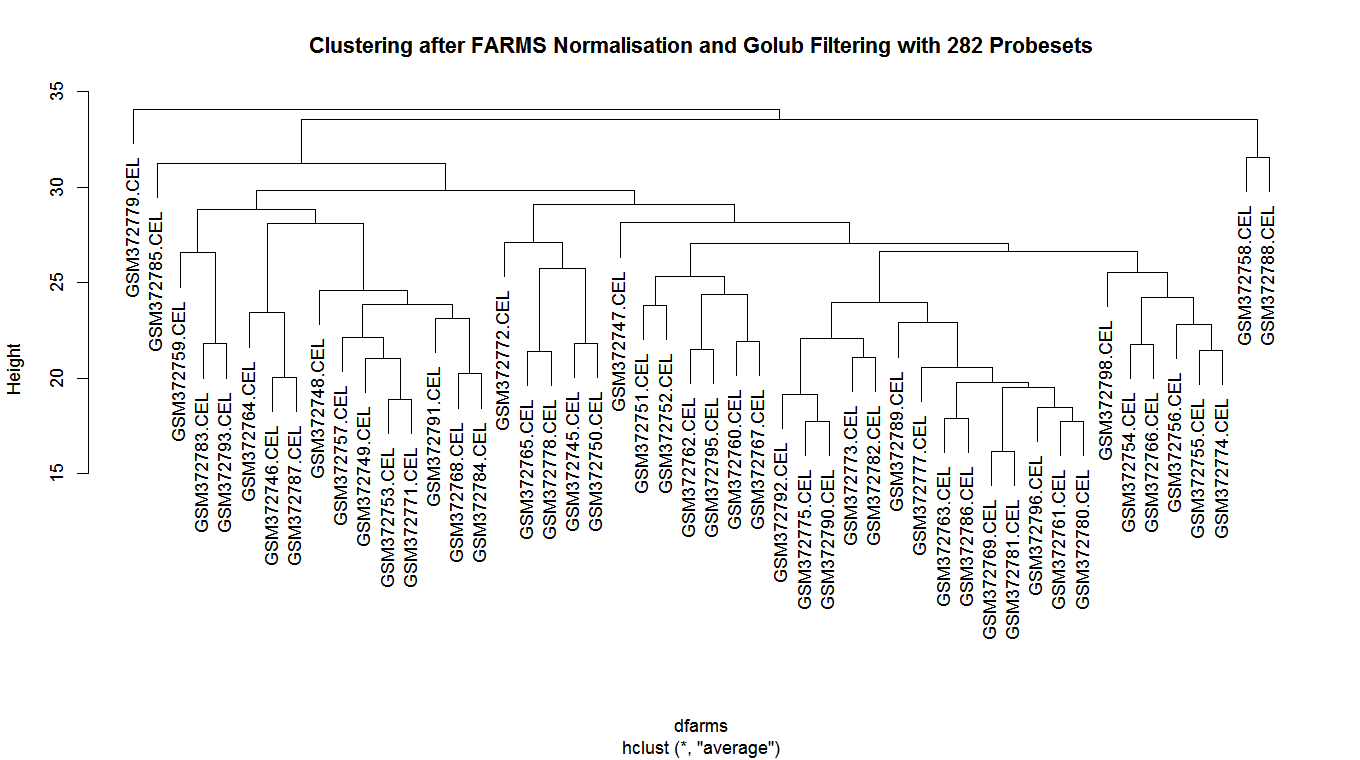

Supplement: Figure S2 — Dendrogram for FARMS normalised data using Golub filtering for 300 probes. (TIFF) [file pone.0050253.s002.tif]

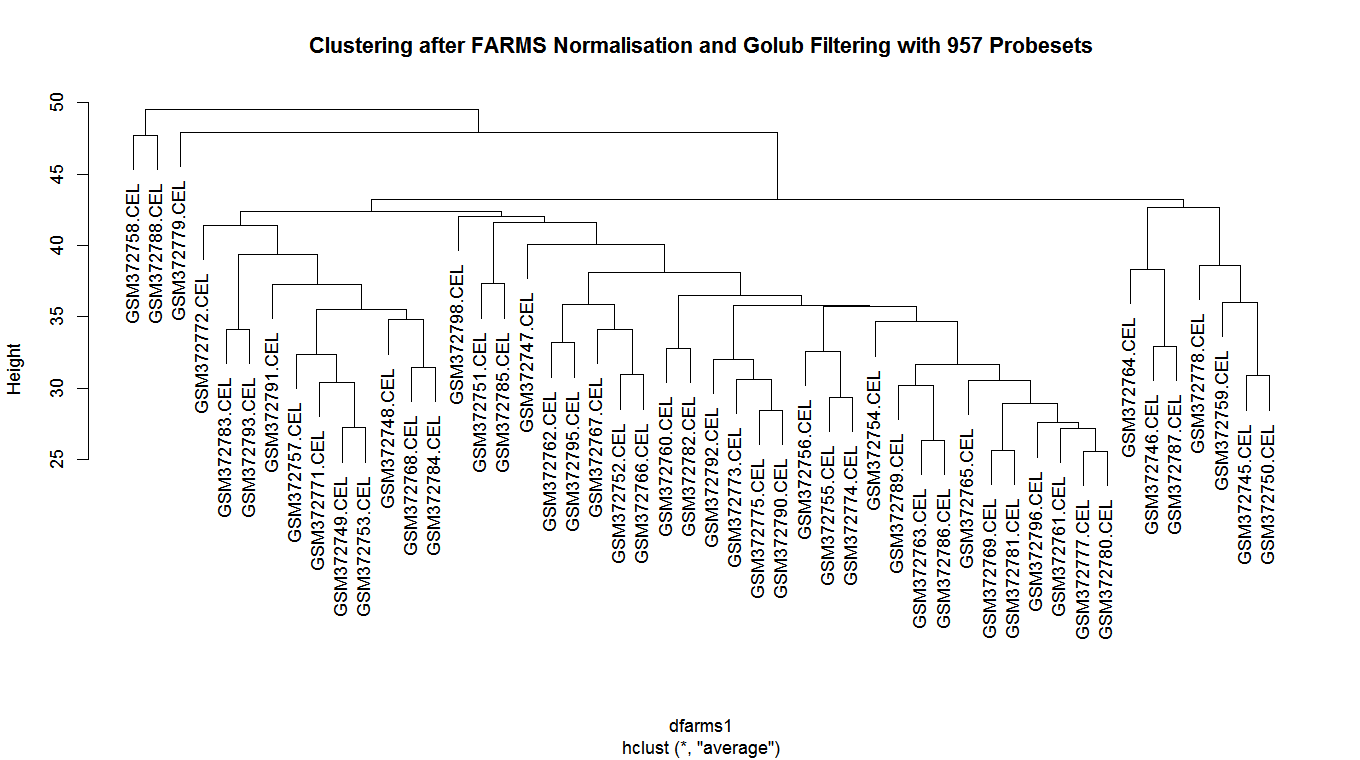

Supplement: Figure S3 — Dendrogram for FARMS normalised data using Golub filtering for 1000 probes. (TIFF) [file pone.0050253.s003.tif]

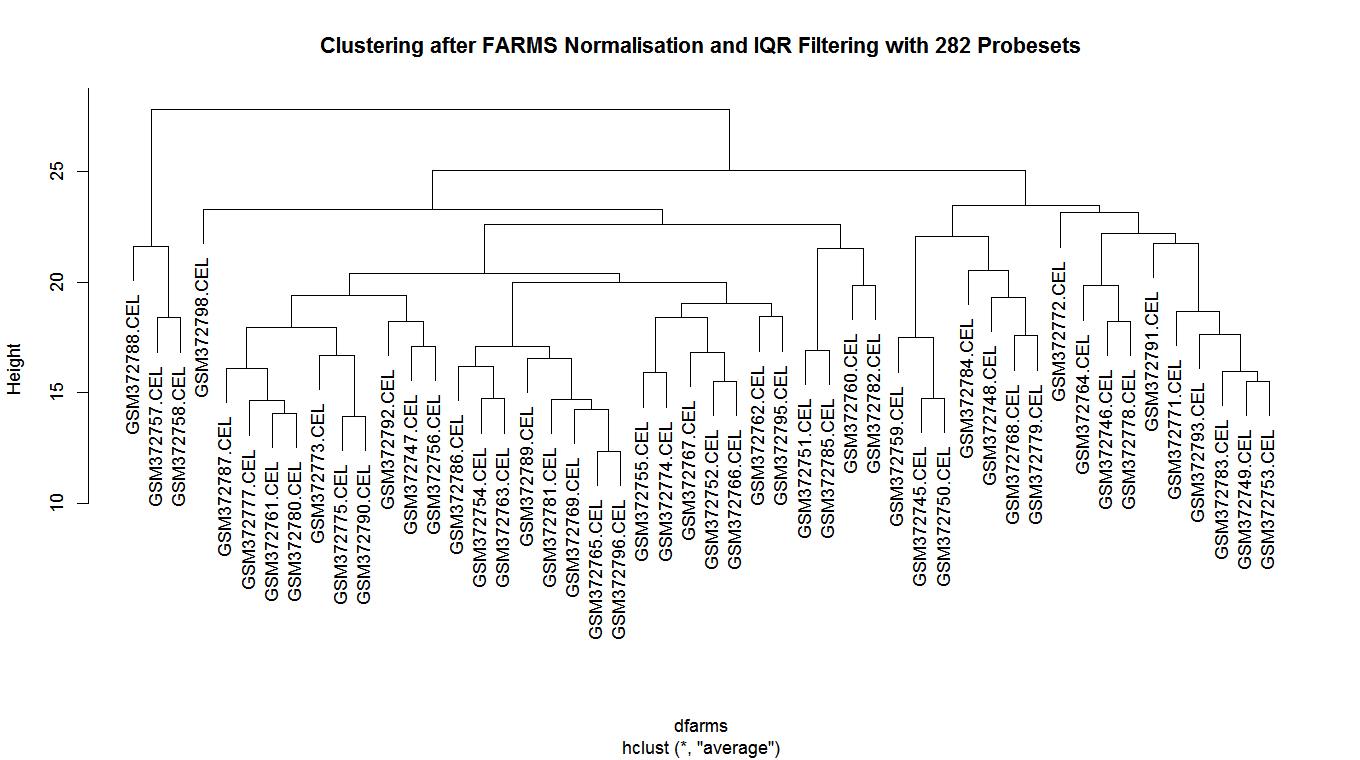

Supplement: Figure S4 — Dendrogram for FARMS normalised data using IQR filtering for 300 probes. (TIFF) [file pone.0050253.s004.tif]

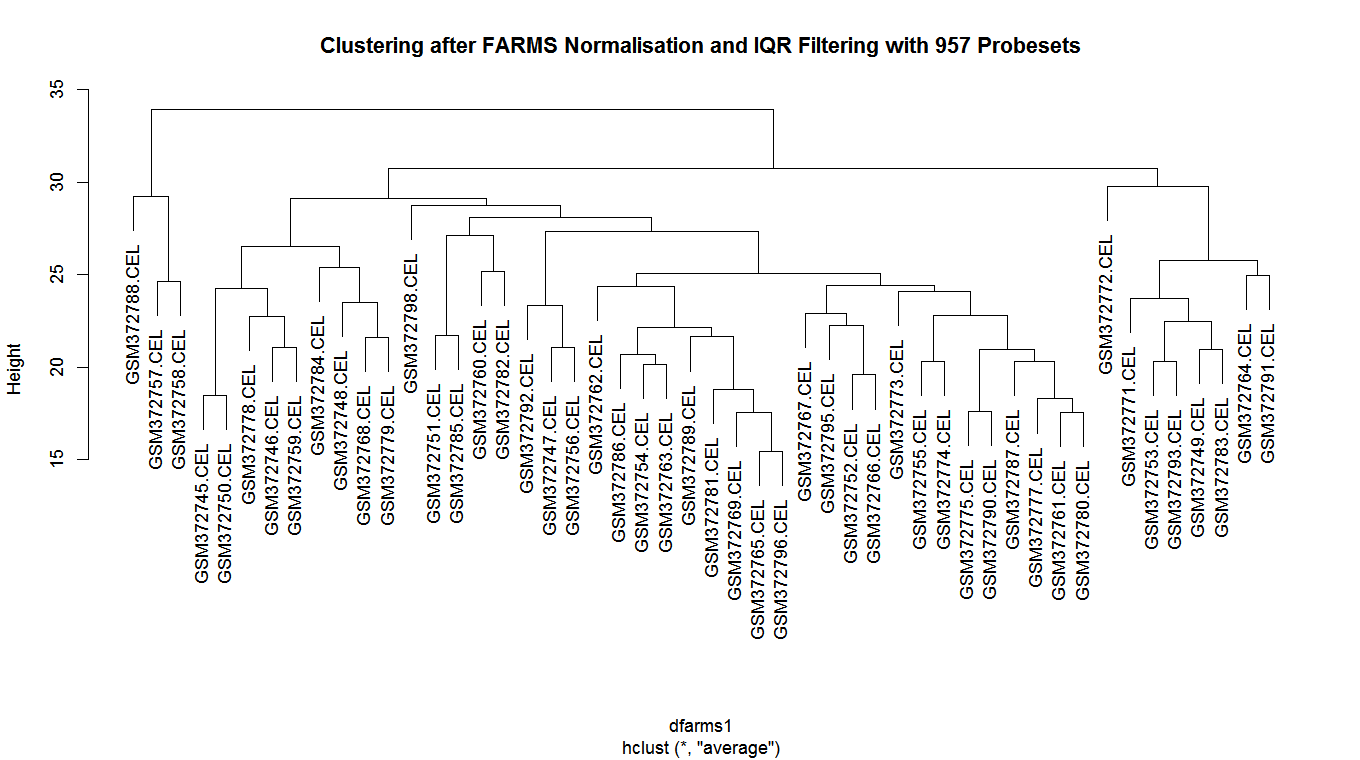

Supplement: Figure S5 — Dendrogram for FARMS normalised data using IQR filtering for 1000 probes. (TIFF) [file pone.0050253.s005.tiff]

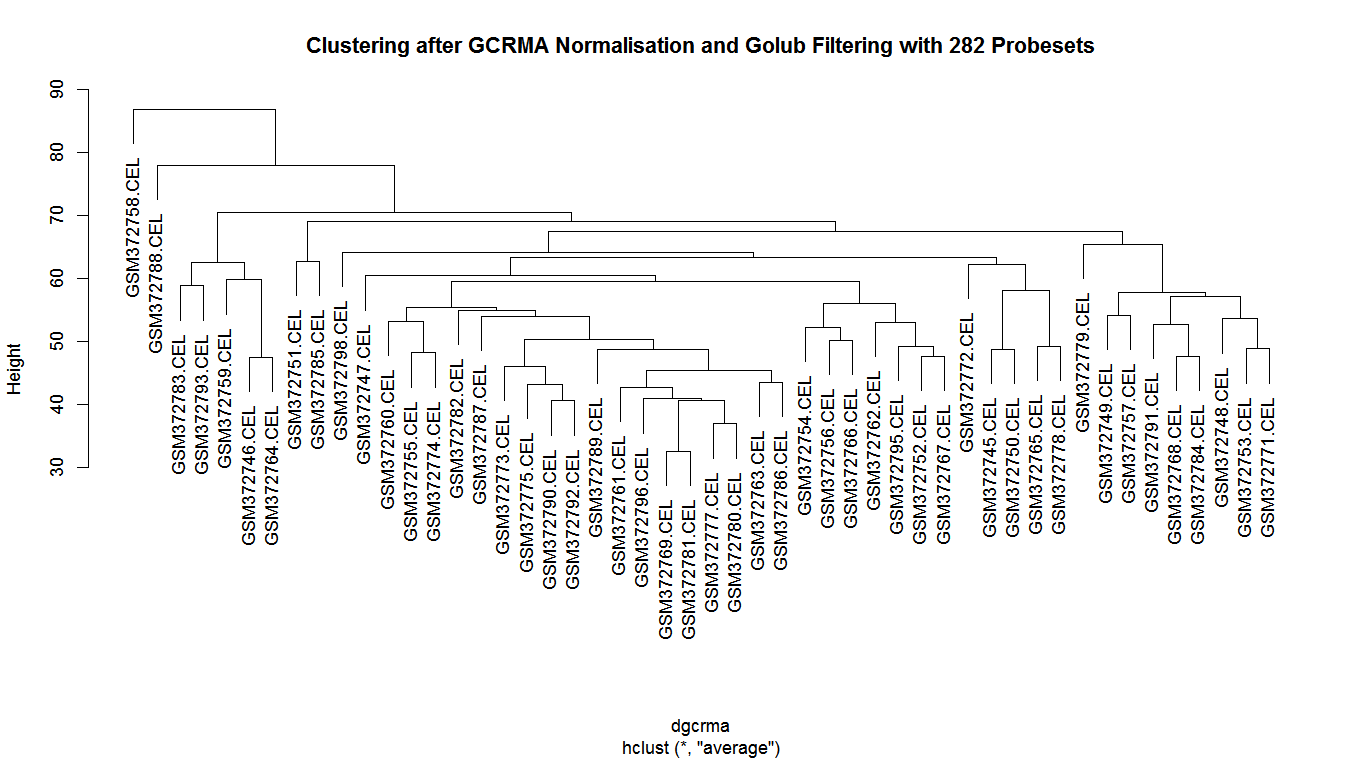

Supplement: Figure S6 — Dendrogram for GCRMA normalised data using Golub filtering for 300 probes. (TIFF) [file pone.0050253.s006.tiff]

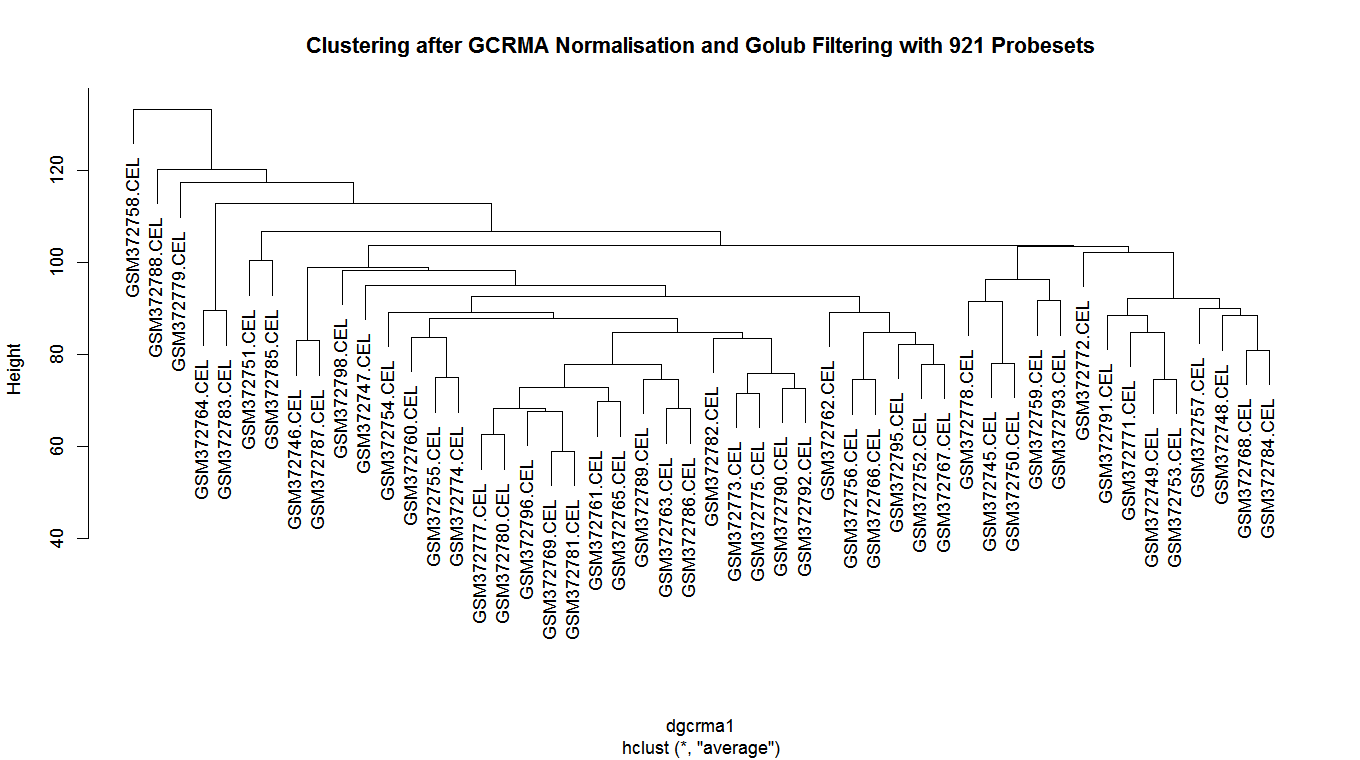

Supplement: Figure S7 — Dendrogram for GCRMA normalised data using Golub filtering for 1000 probes. (TIFF) [file pone.0050253.s007.tiff]

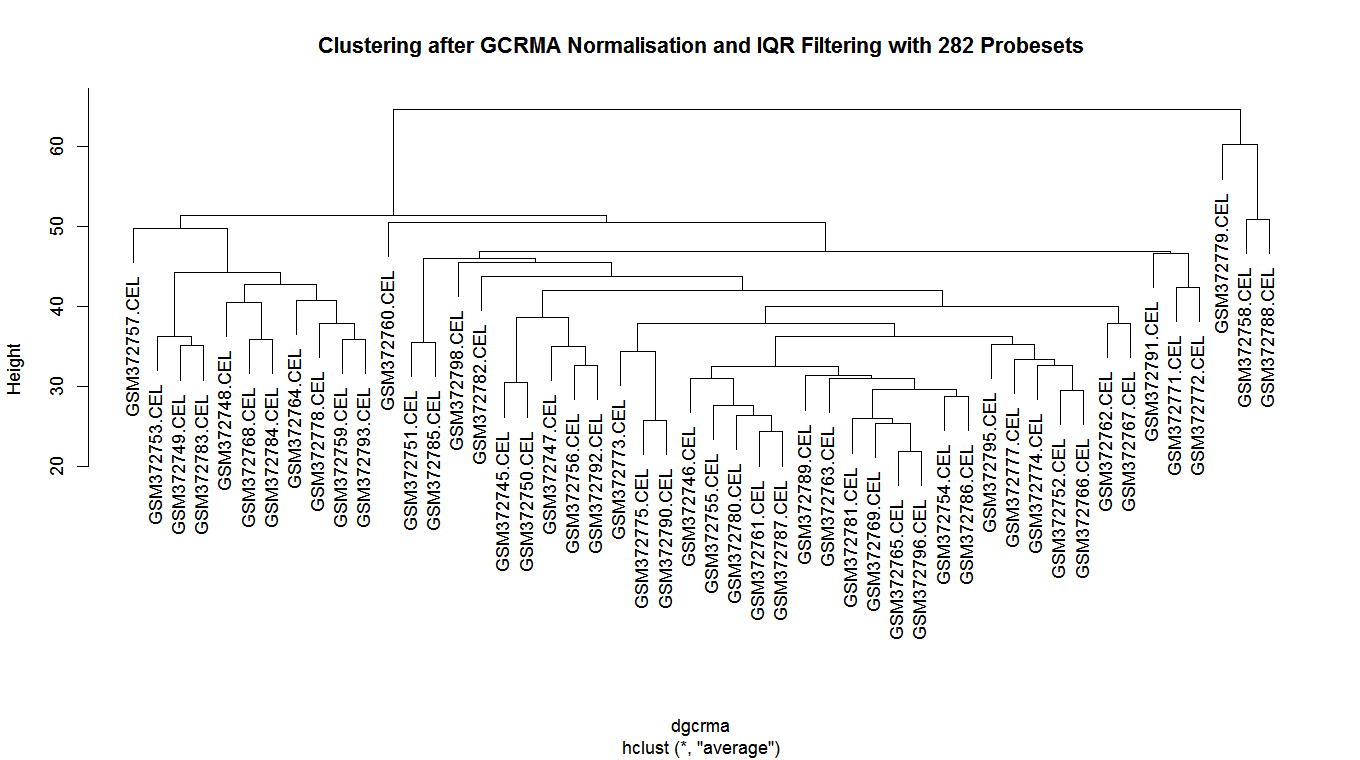

Supplement: Figure S8 — Dendrogram for GCRMA normalised data using IQR filtering for 300 probes. (TIFF) [file pone.0050253.s008.tiff]

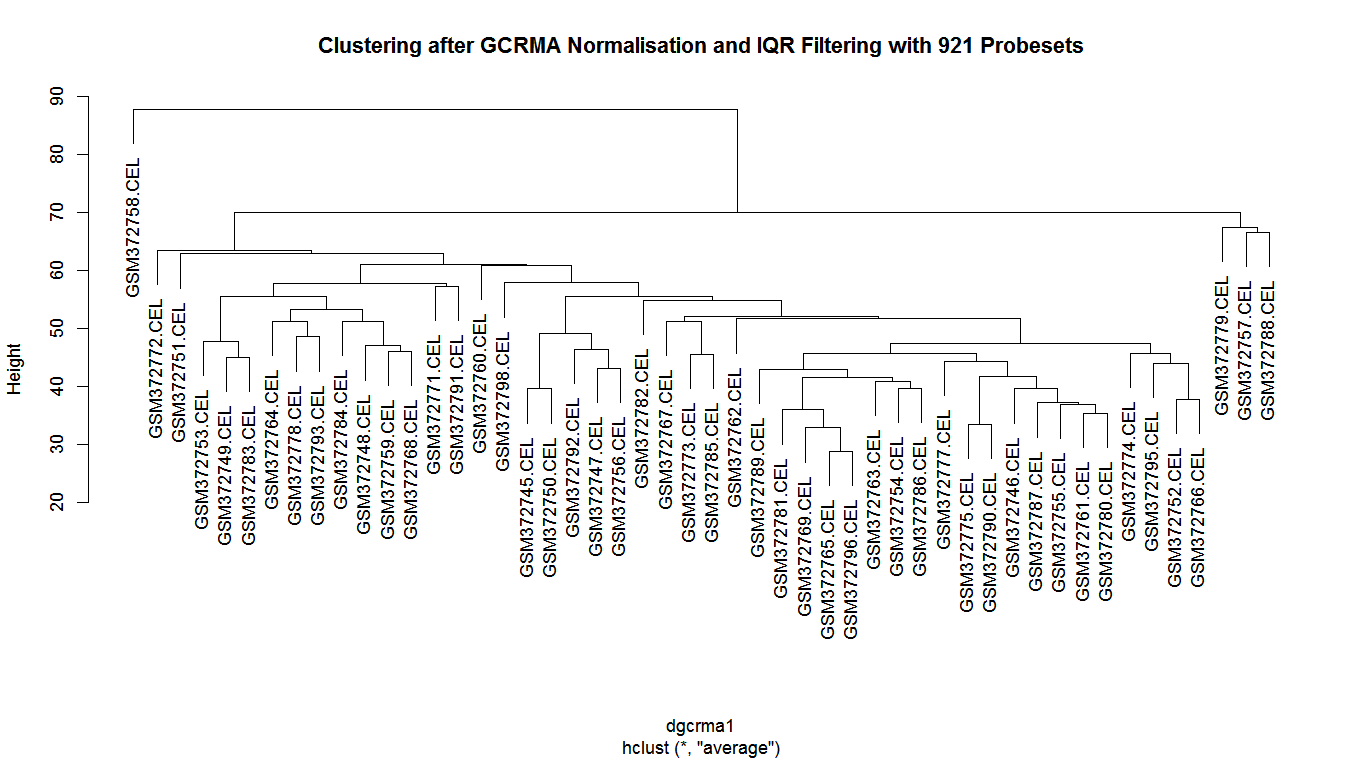

Supplement: Figure S9 — Dendrogram for GCRMA normalised data using IQR filtering for 1000 probes. (TIFF) [file pone.0050253.s009.tiff]

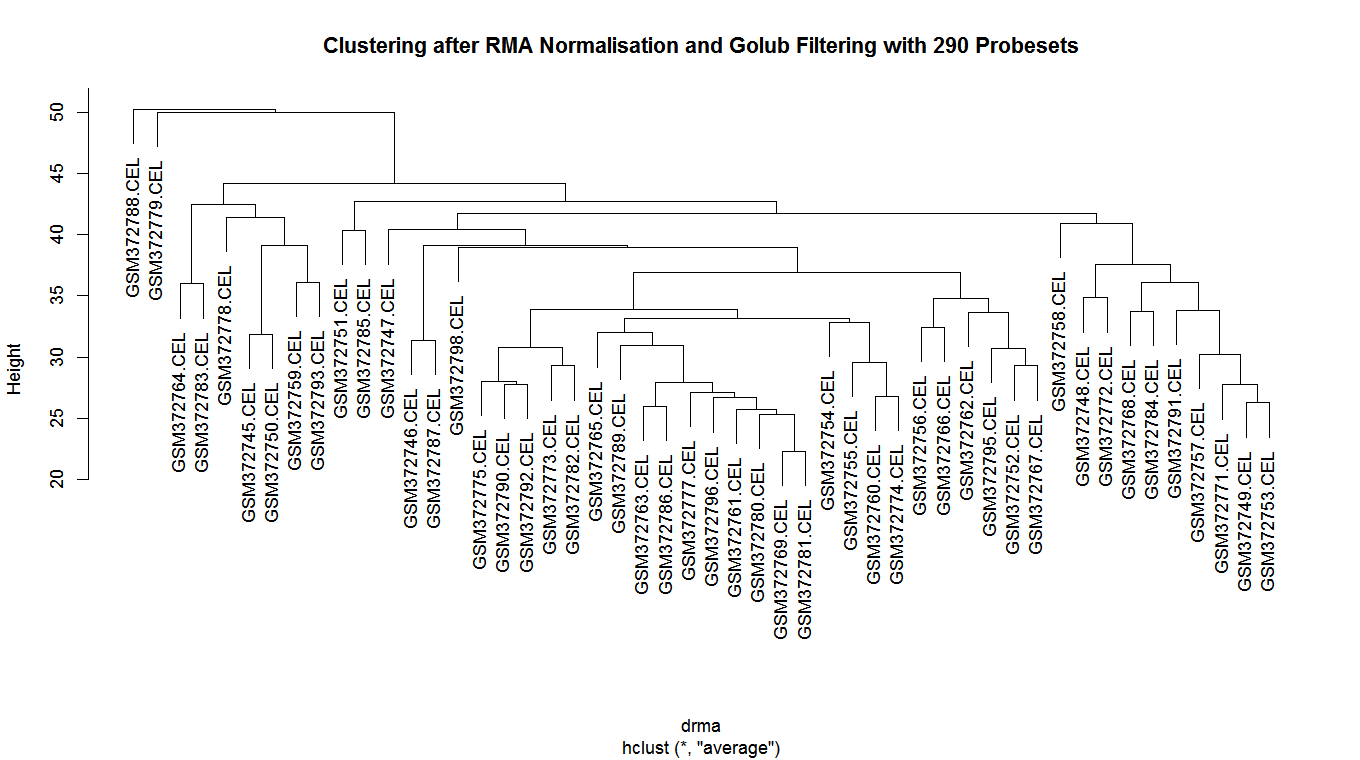

Supplement: Figure S10 — Dendrogram for RMA normalised data using Golub filtering for 300 probes. (TIFF) [file pone.0050253.s010.tiff]

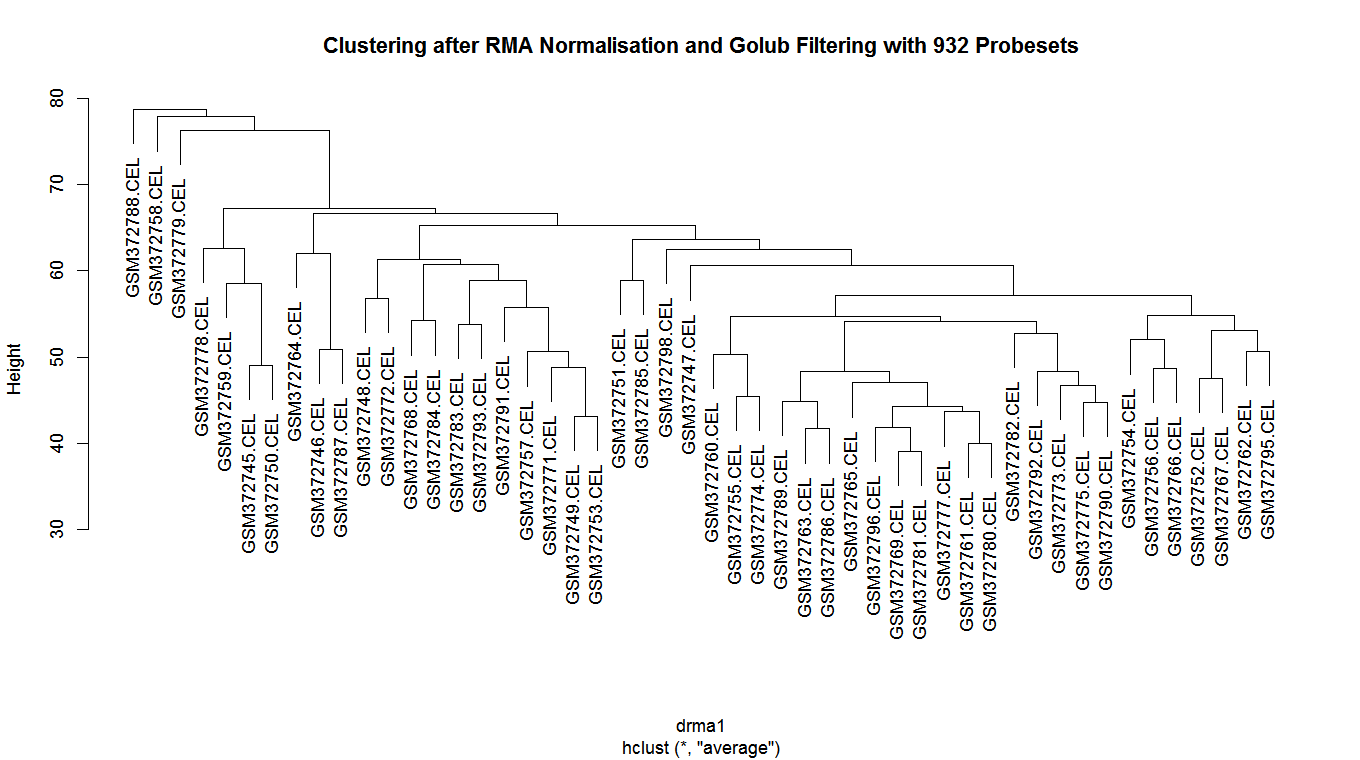

Supplement: Figure S11 — Dendrogram for RMA normalised data using Golub filtering for 1000 probes. (TIFF) [file pone.0050253.s011.tiff]

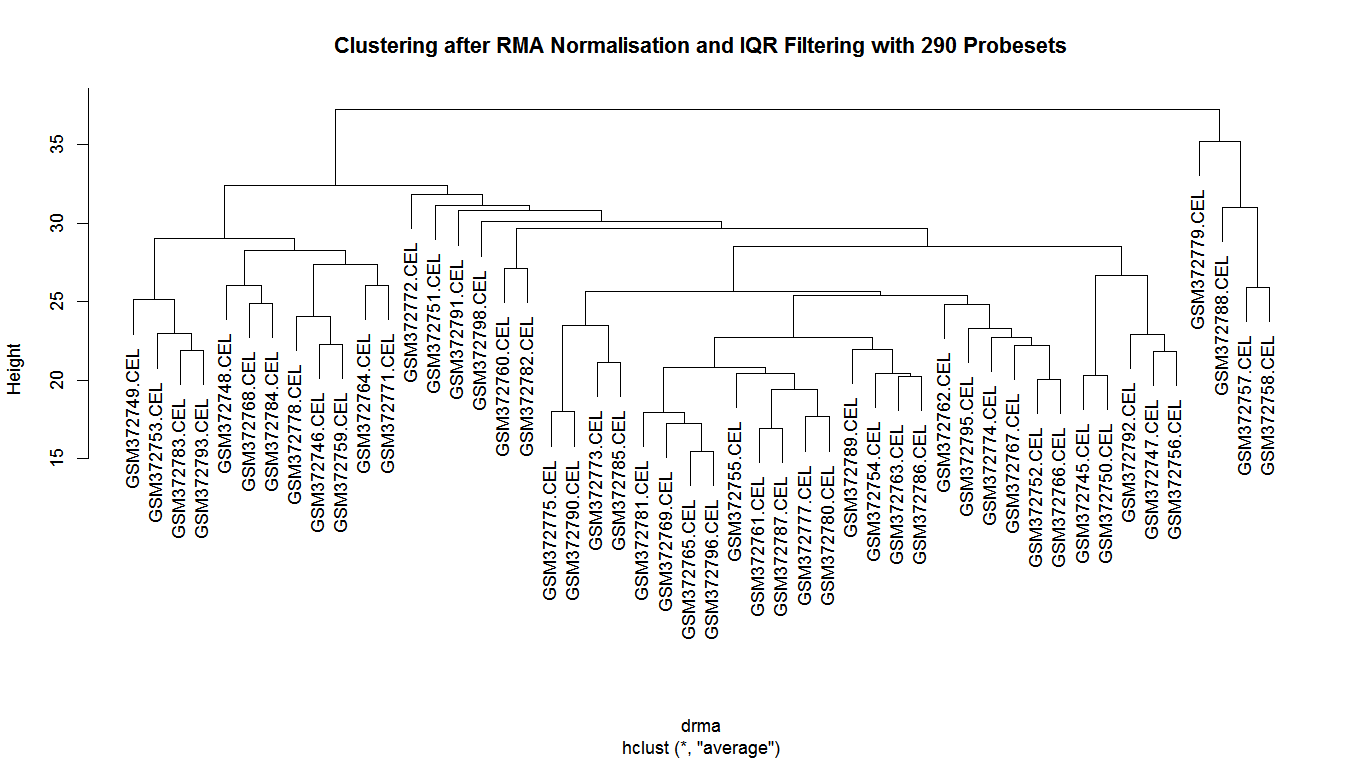

Supplement: Figure S12 — Dendrogram for RMA normalised data using IQR filtering for 300 probes. (TIFF) [file pone.0050253.s012.tiff]

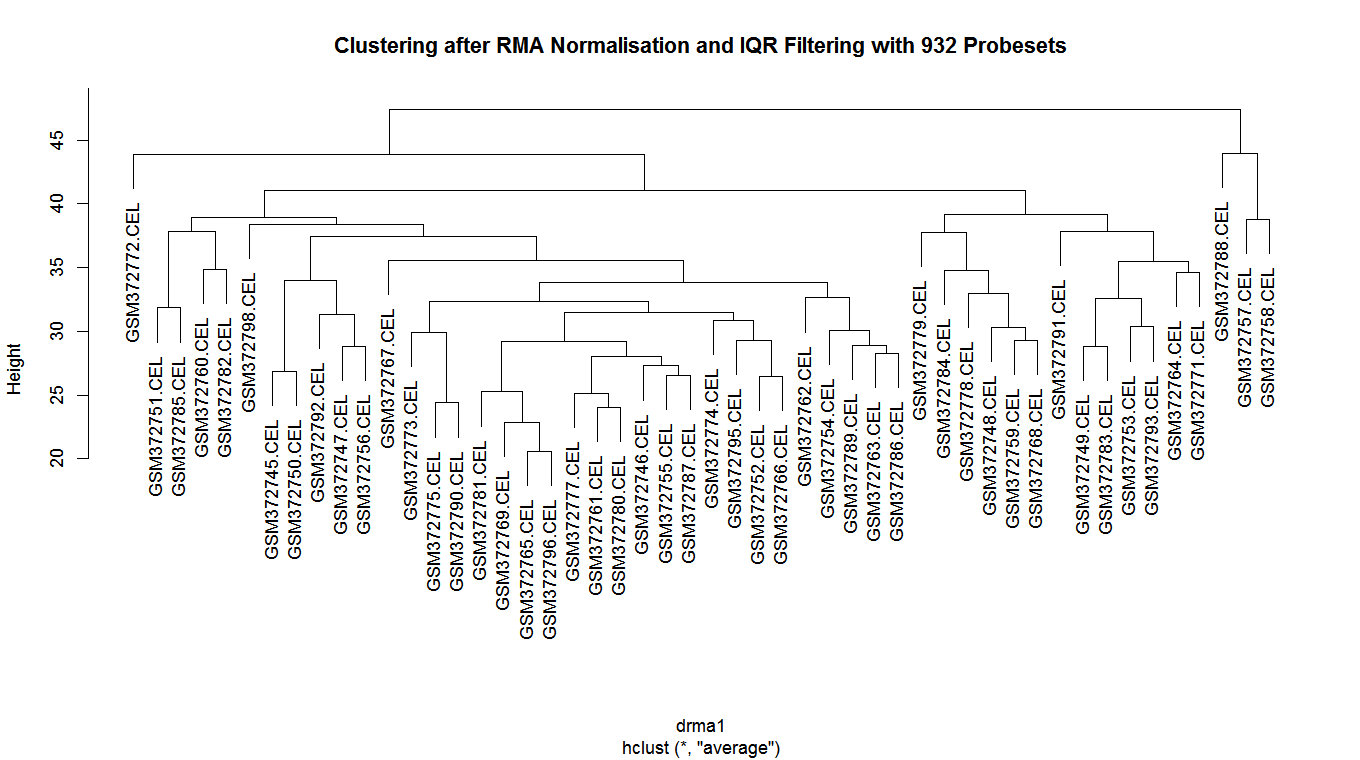

Supplement: Figure S13 — Dendrogram for RMA normalised data using IQR filtering for 1000 probes. (TIFF) [file pone.0050253.s013.tiff]

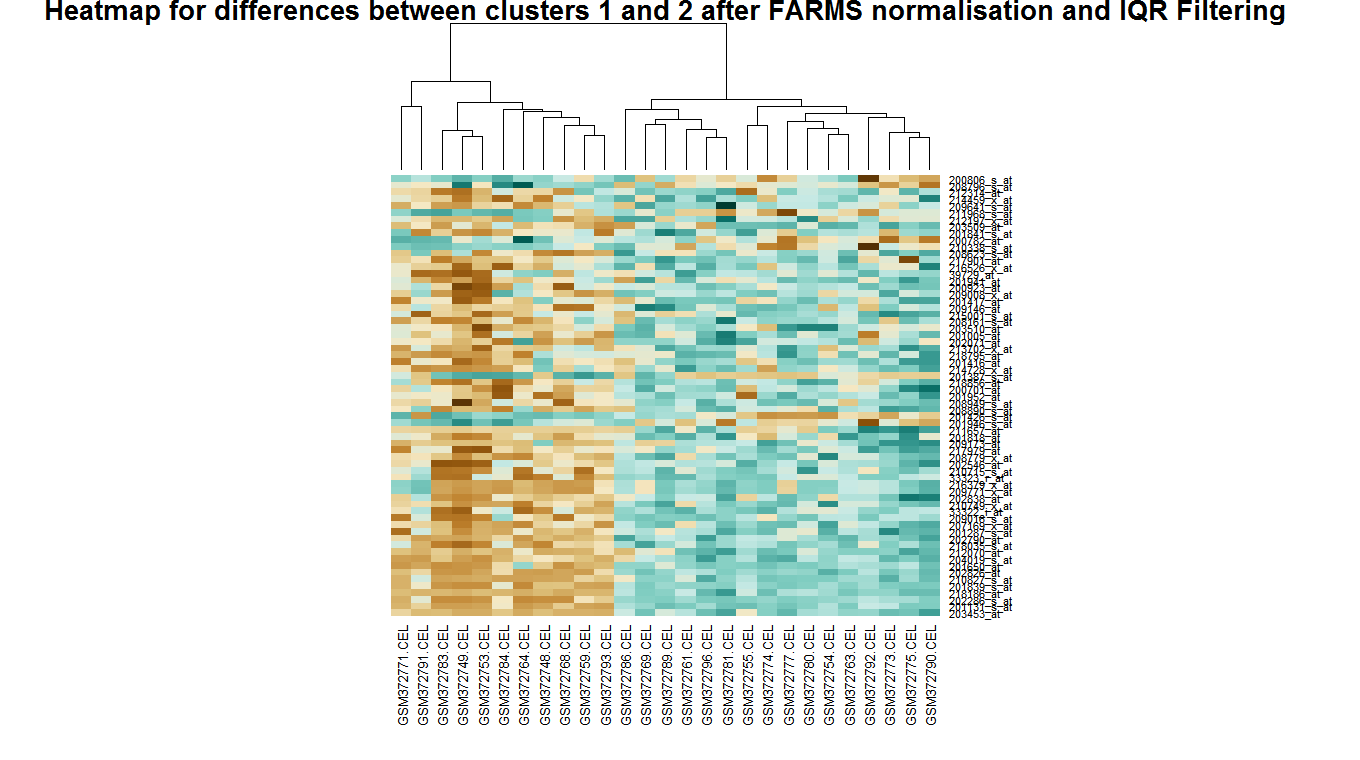

Supplement: Figure S14 — Heatmap for the differentially expressed genes between clusters 1 and 2 after Normalisation with FARMS and IQR filtering for 300 probes. (TIFF) [file pone.0050253.s014.tiff]

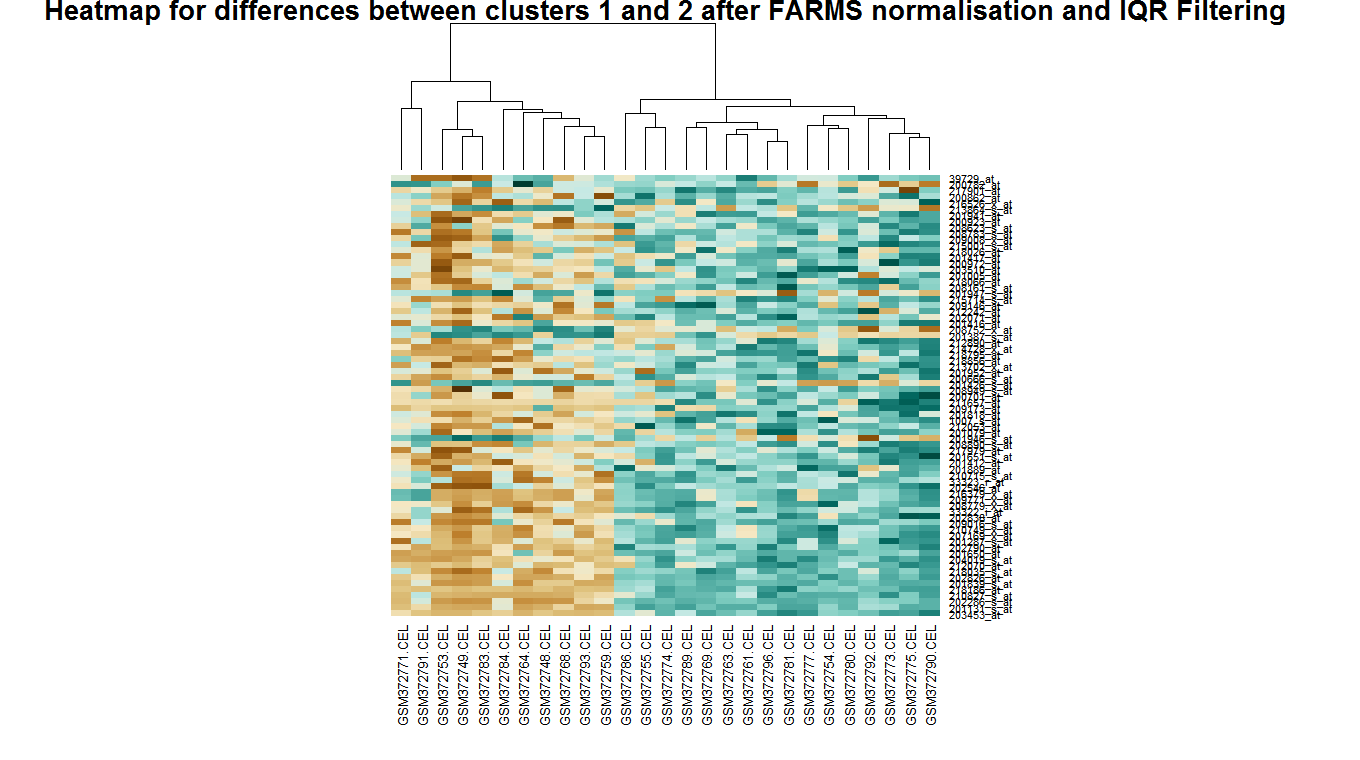

Supplement: Figure S15 — Heatmap for the differentially expressed genes between clusters 1 and 2 after Normalisation with FARMS and IQR filtering for 1000 probes. (TIFF) [file pone.0050253.s015.tiff]

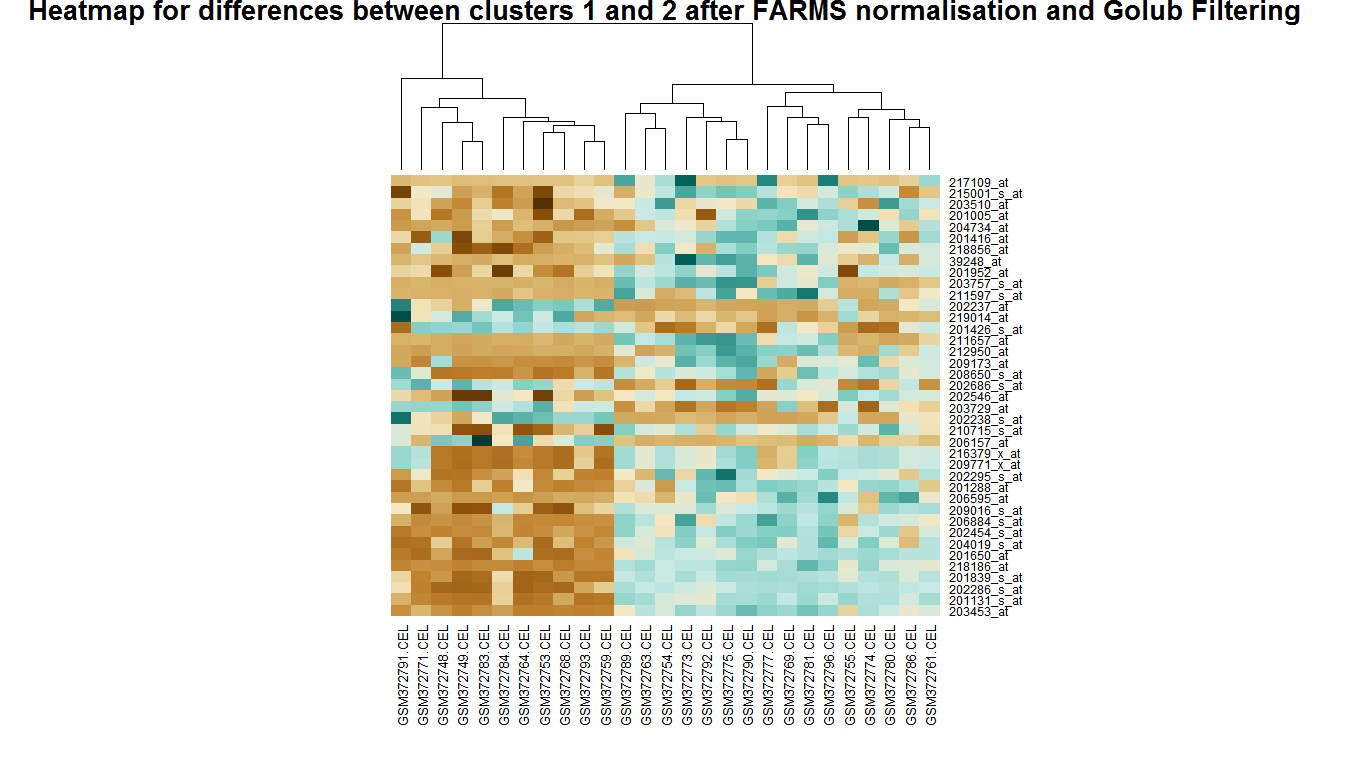

Supplement: Figure S16 — Heatmap for the differentially expressed genes between clusters 1 and 2 after Normalisation with FARMS and Golub filtering for 300 probes. (TIFF) [file pone.0050253.s016.tiff]

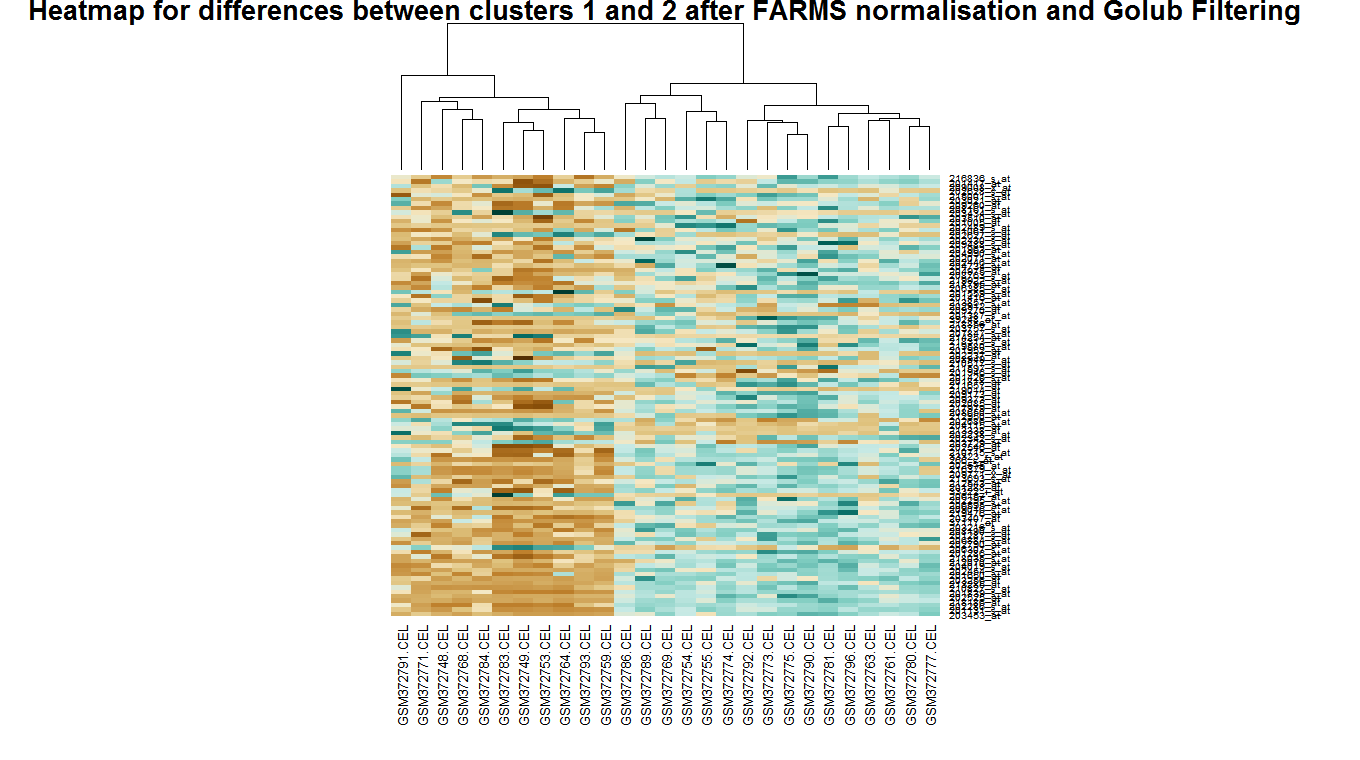

Supplement: Figure S17 — Heatmap for the differentially expressed genes between clusters 1 and 2 after Normalisation with FARMS and Golub filtering for 1000 probes. (TIFF) [file pone.0050253.s017.tiff]

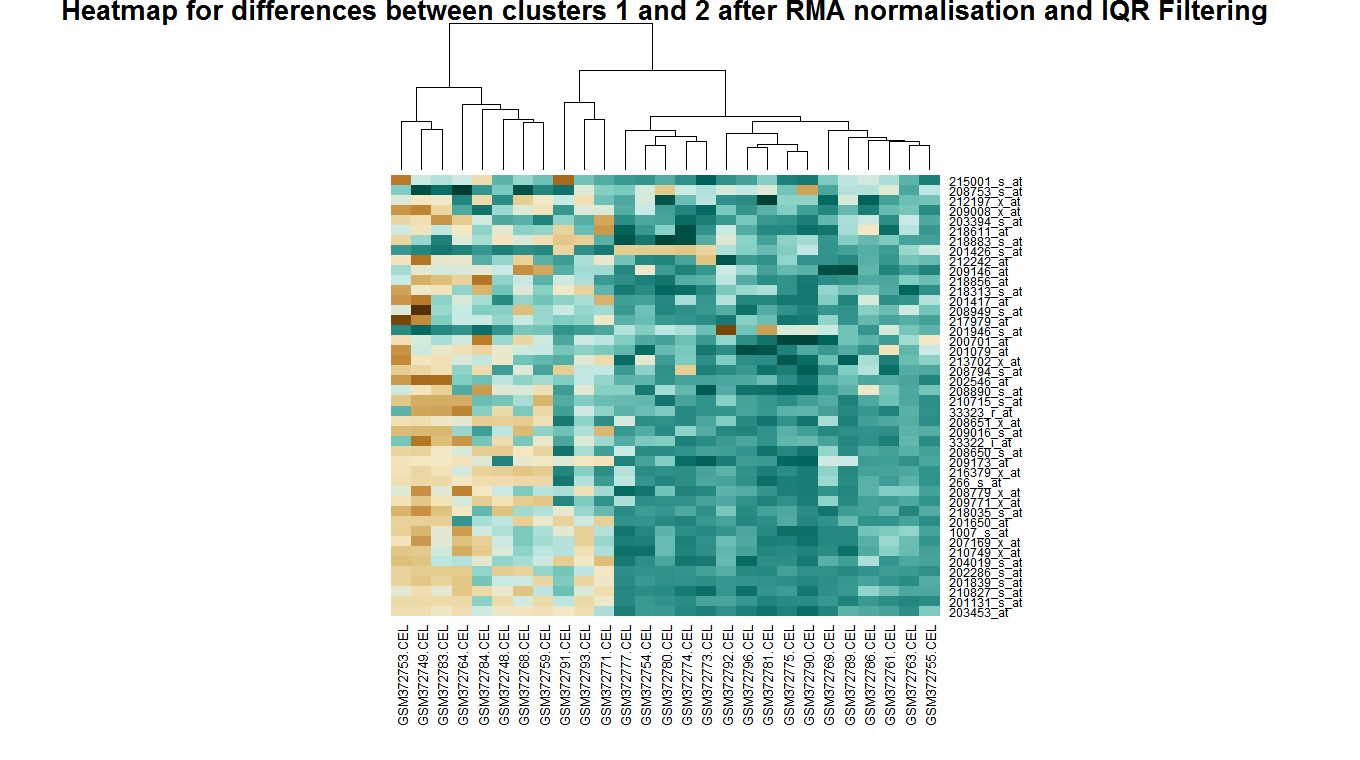

Supplement: Figure S18 — Heatmap for the differentially expressed genes between clusters 1 and 2 after Normalisation with GCRMA and IQR filtering for 300 probes. (TIFF) [file pone.0050253.s018.tiff]

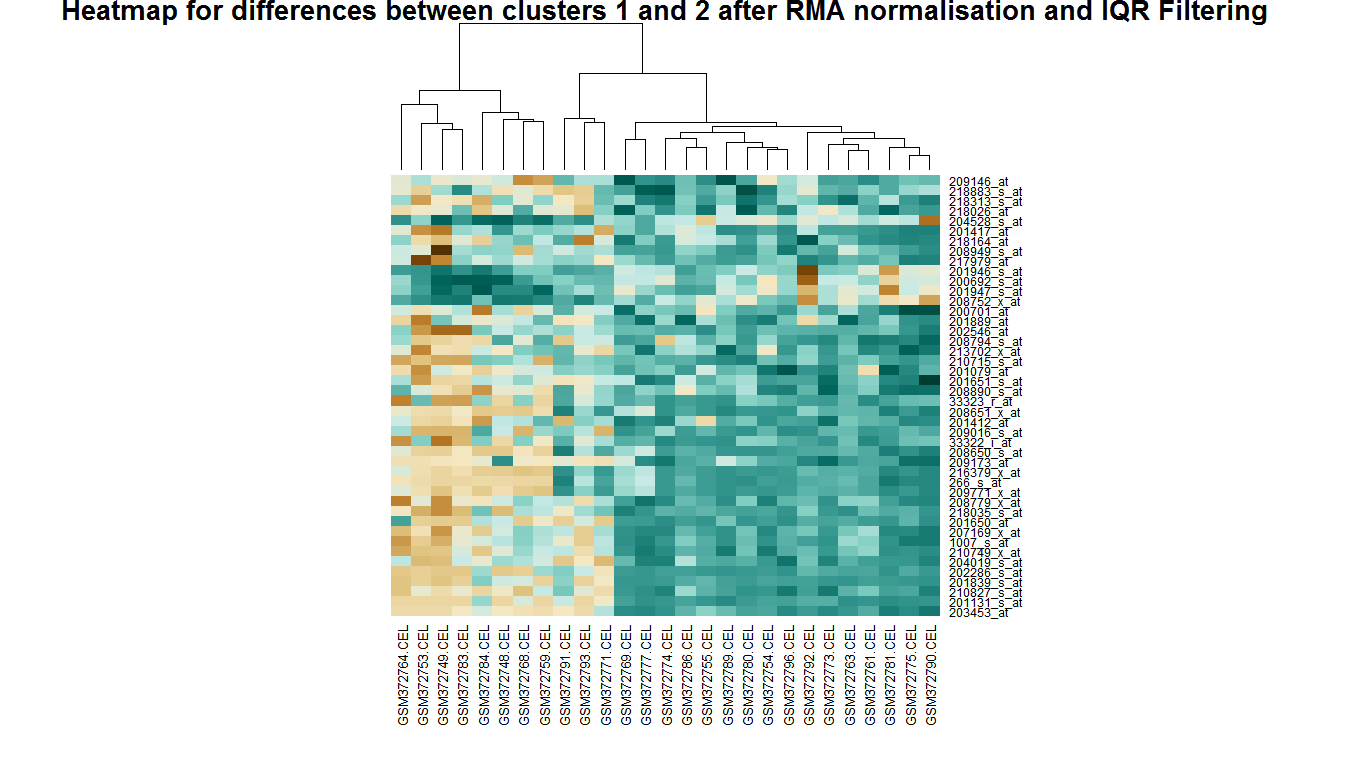

Supplement: Figure S19 — Heatmap for the differentially expressed genes between clusters 1 and 2 after Normalisation with GCRMA and IQR filtering for 1000 probes. (TIFF) [file pone.0050253.s019.tiff]

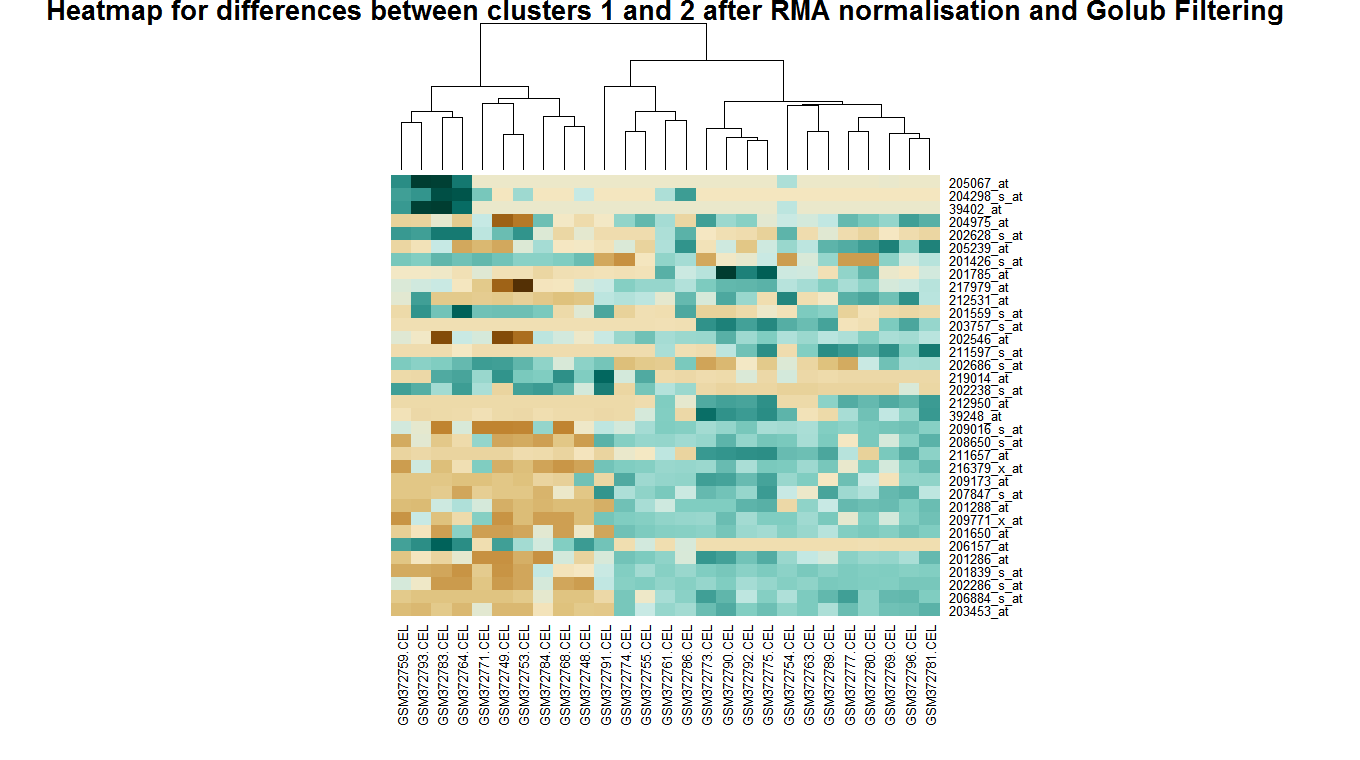

Supplement: Figure S20 — Heatmap for the differentially expressed genes between clusters 1 and 2 after Normalisation with GCRMA and Golub filtering for 300 probes. (TIFF) [file pone.0050253.s020.tiff]

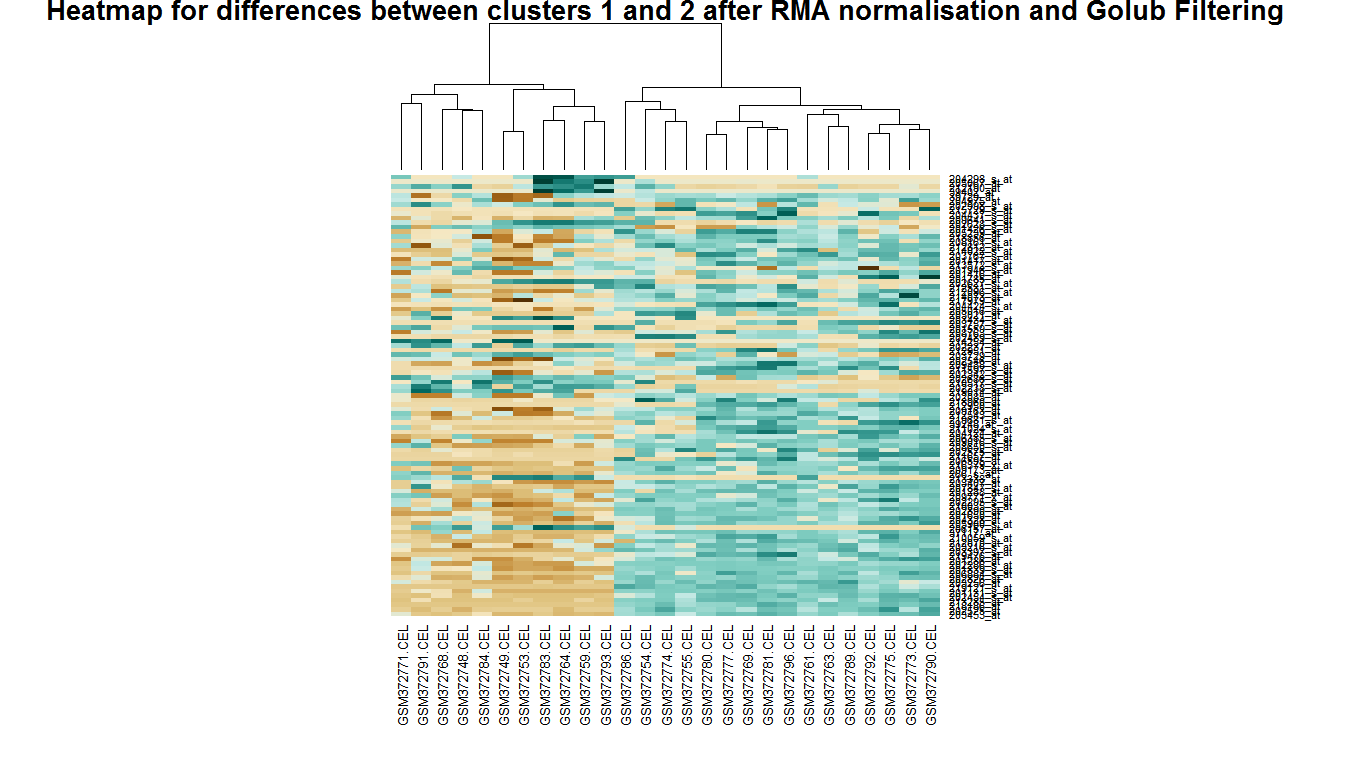

Supplement: Figure S21 — Heatmap for the differentially expressed genes between clusters 1 and 2 after Normalisation with GCRMA and Golub filtering for 1000 probes. (TIFF) [file pone.0050253.s021.tiff]

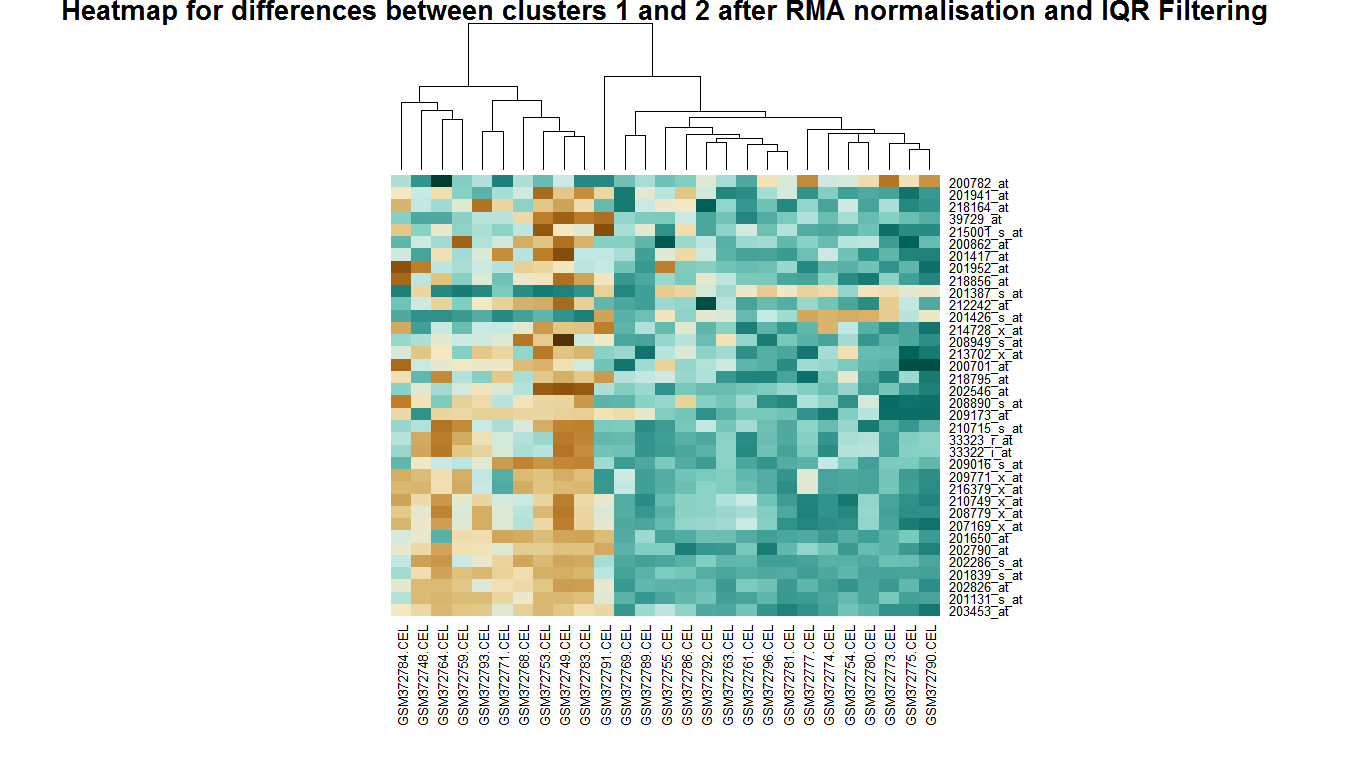

Supplement: Figure S22 — Heatmap for the differentially expressed genes between clusters 1 and 2 after Normalisation with RMA and IQR filtering for 300 probes. (TIFF) [file pone.0050253.s022.tiff]

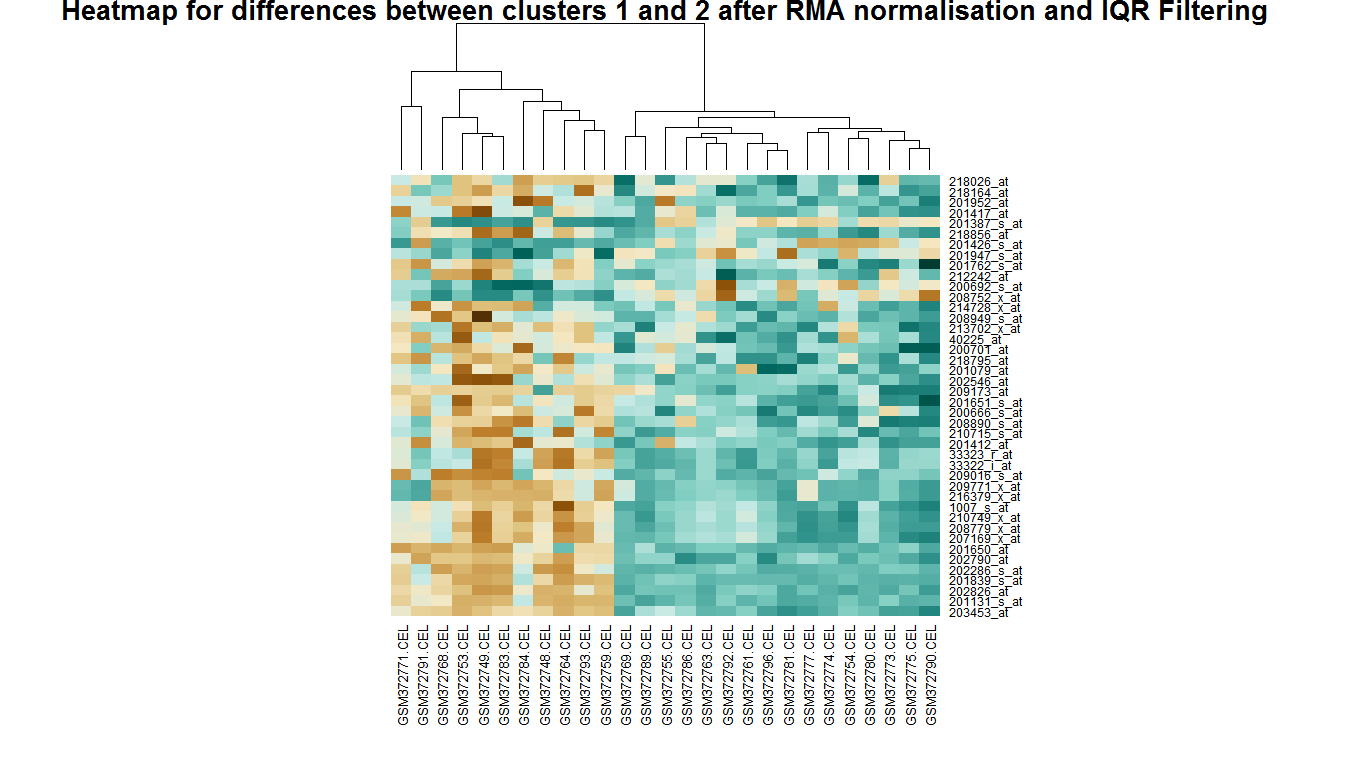

Supplement: Figure S23 — Heatmap for the differentially expressed genes between clusters 1 and 2 after Normalisation with RMA and IQR filtering for 1000 probes. (TIFF) [file pone.0050253.s023.tiff]

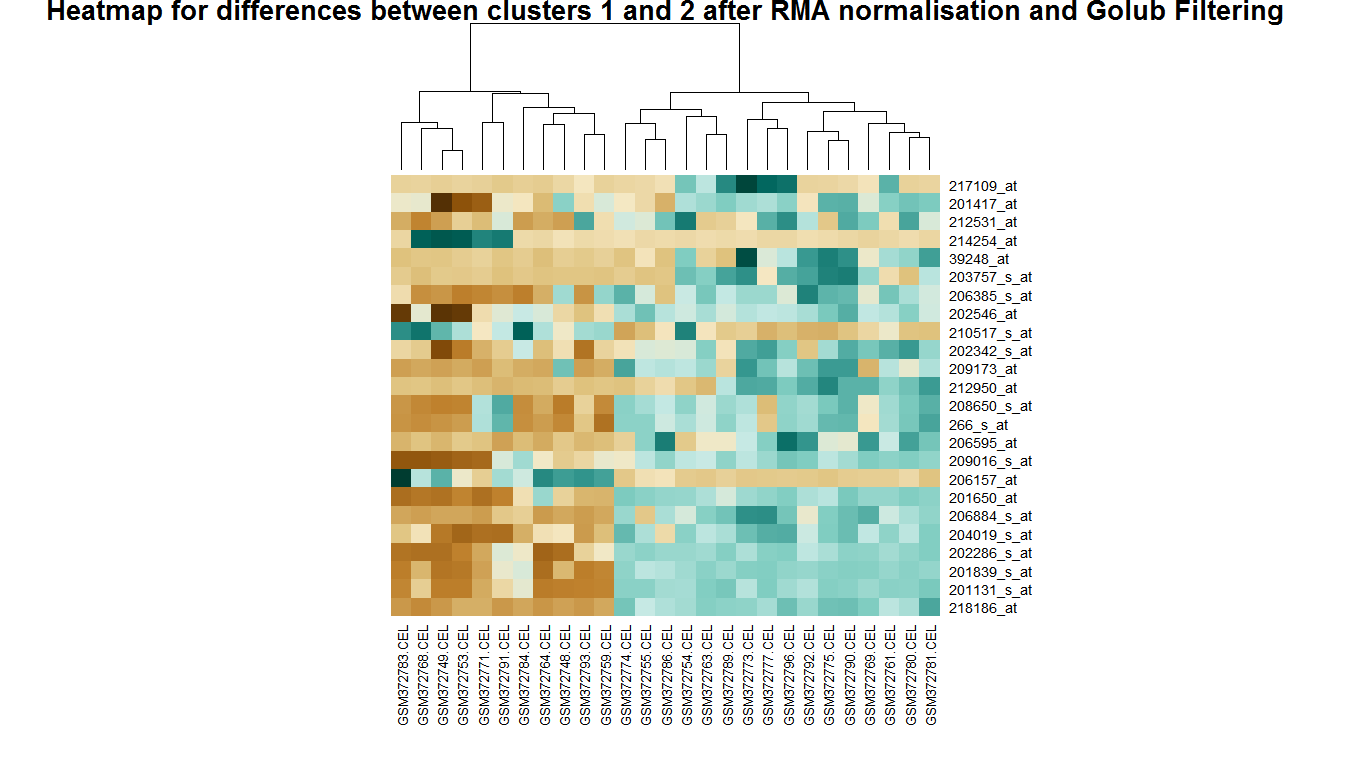

Supplement: Figure S24 — Heatmap for the differentially expressed genes between clusters 1 and 2 after Normalisation with RMA and Golub filtering for 300 probes. (TIFF) [file pone.0050253.s024.tiff]

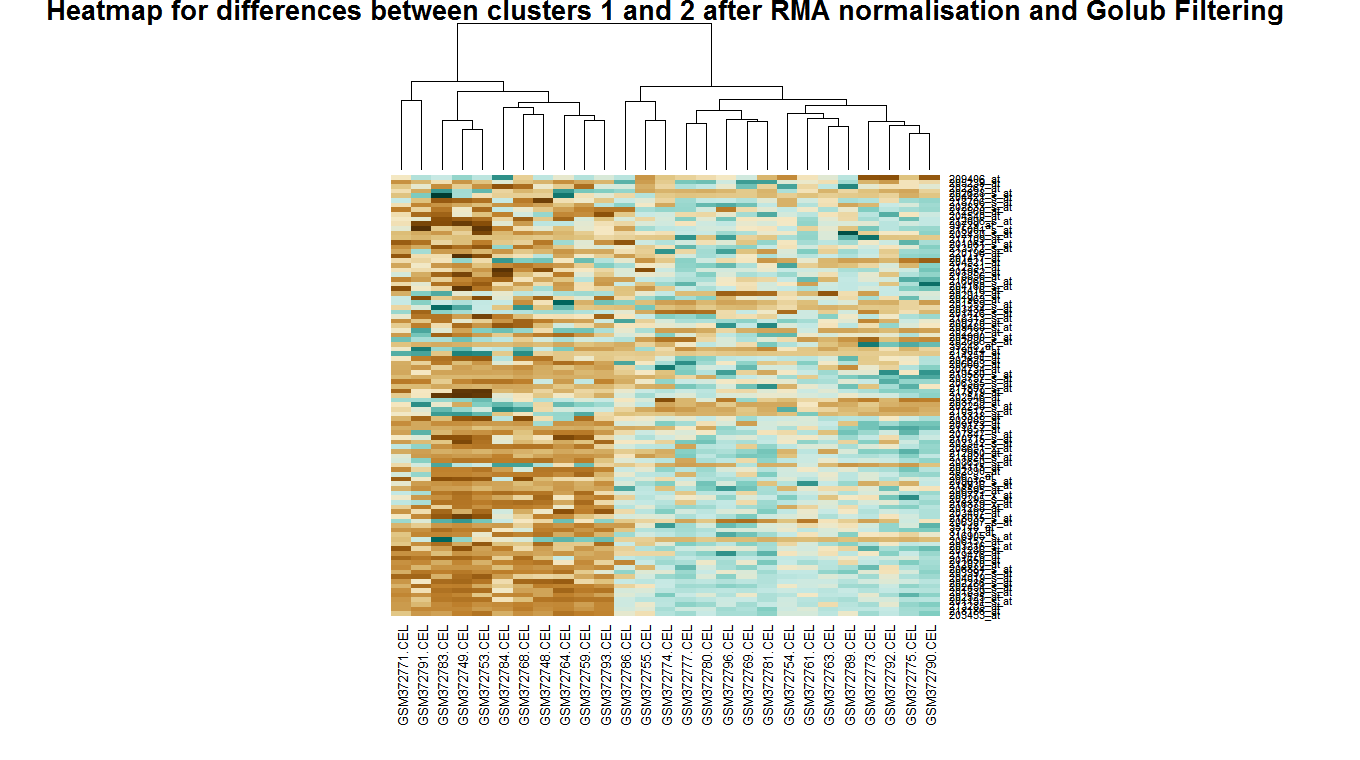

Supplement: Figure S25 — Heatmap for the differentially expressed genes between clusters 1 and 2 after Normalisation with RMA and Golub filtering for 1000 probes. (TIFF) [file pone.0050253.s025.tiff]

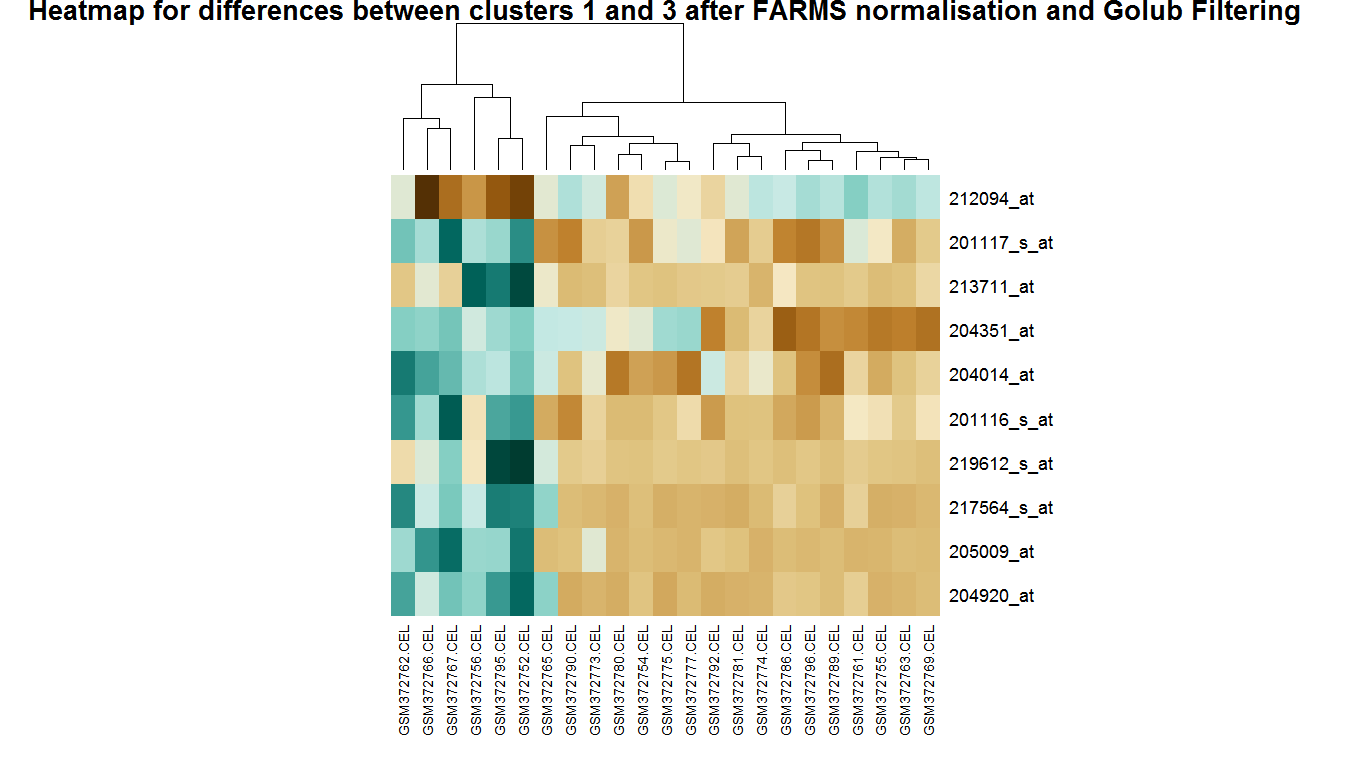

Supplement: Figure S26 — Heatmap for the differentially expressed genes between clusters 1 and 3 after Normalisation with FARMS and Golub filtering for 300 probes (TIFF) [file pone.0050253.s026.tiff]

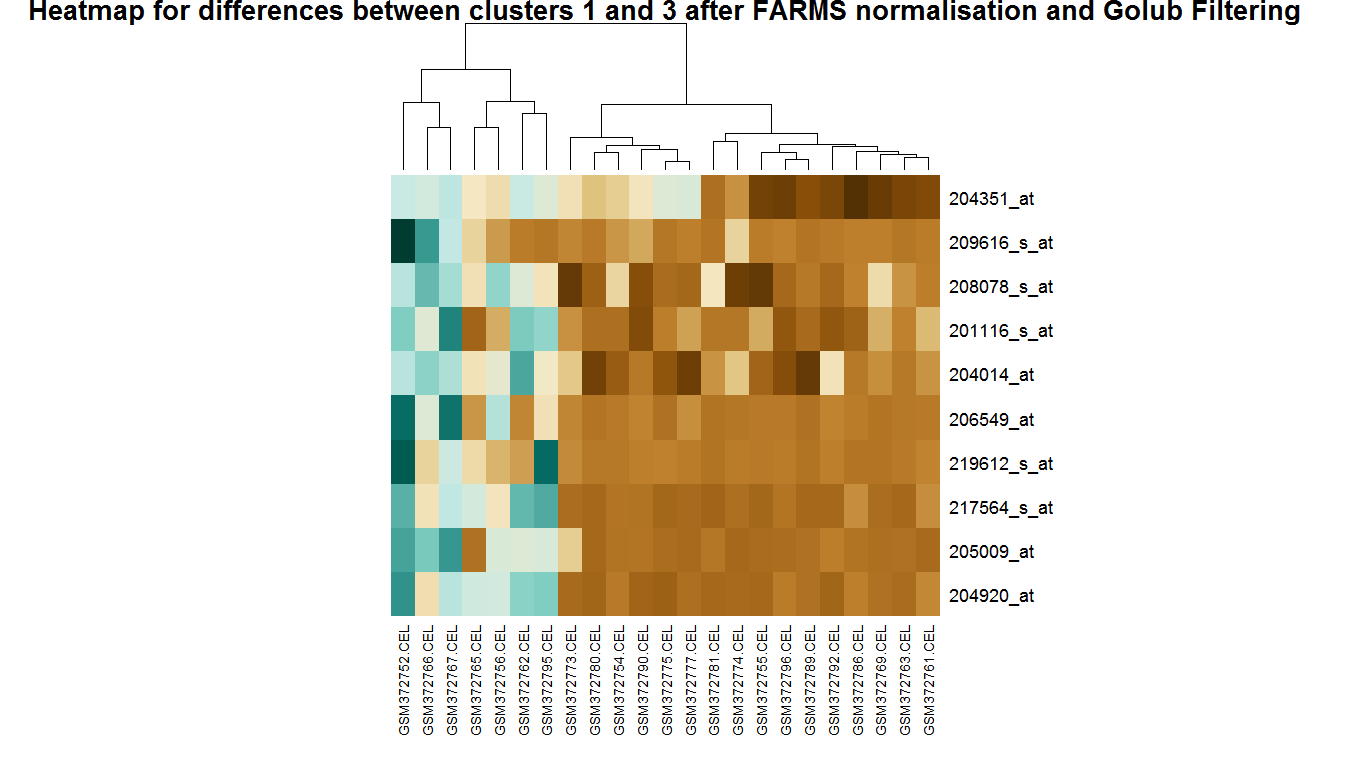

Supplement: Figure S27 — Heatmap for the differentially expressed genes between clusters 1 and 3 after Normalisation with FARMS and Golub filtering for 1000 probes (TIFF) [file pone.0050253.s027.tiff]

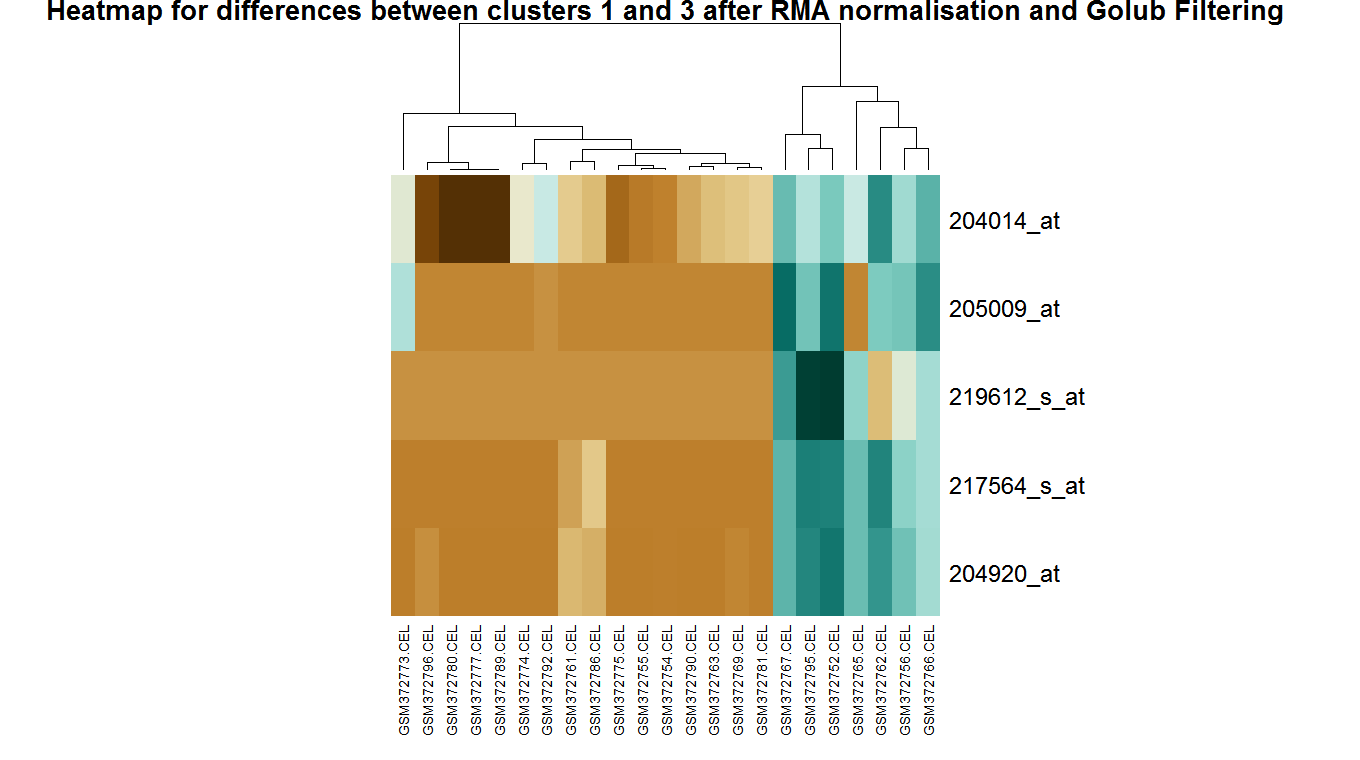

Supplement: Figure S28 — Heatmap for the differentially expressed genes between clusters 1 and 3 after Normalisation with GCRMA and Golub filtering for 300 probes (TIFF) [file pone.0050253.s028.tiff]

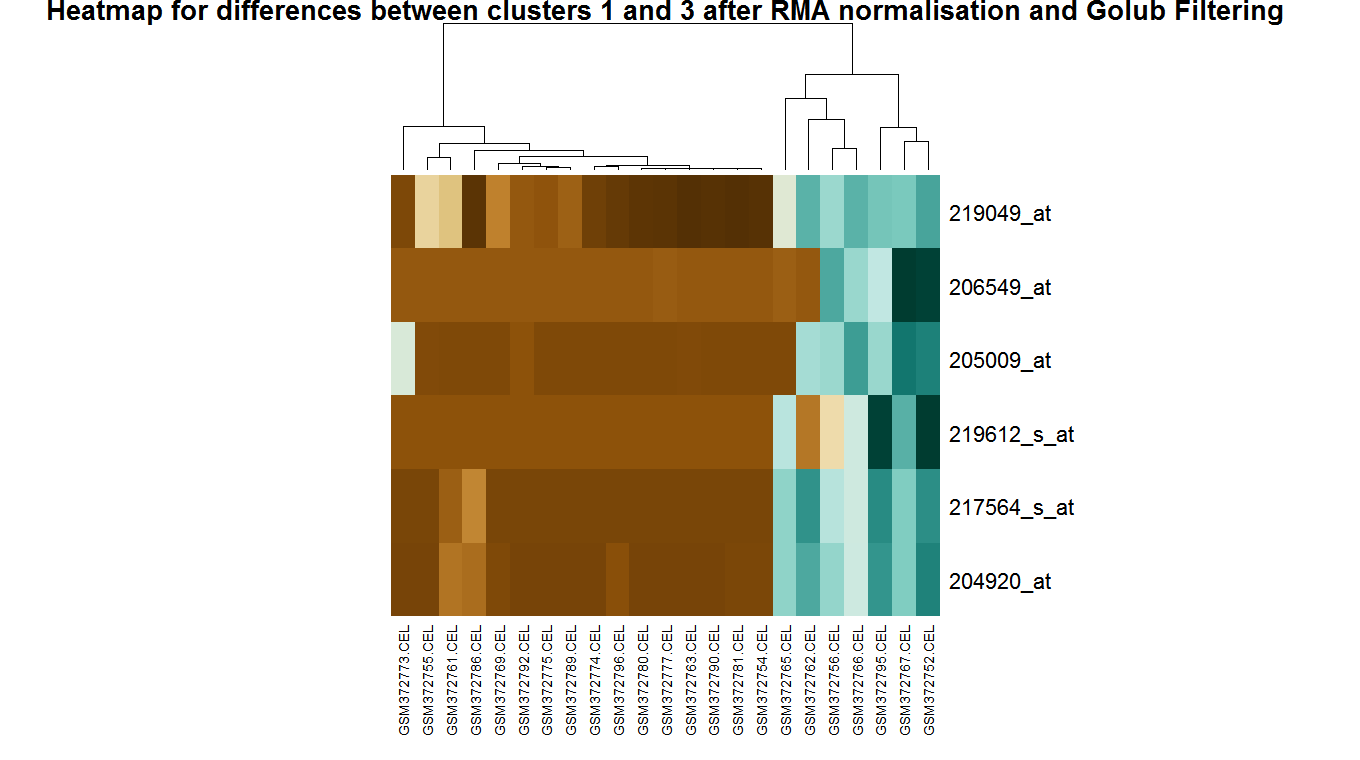

Supplement: Figure S29 — Heatmap for the differentially expressed genes between clusters 1 and 3 after Normalisation with GCRMA and Golub filtering for 1000 probes (TIFF) [file pone.0050253.s029.tiff]

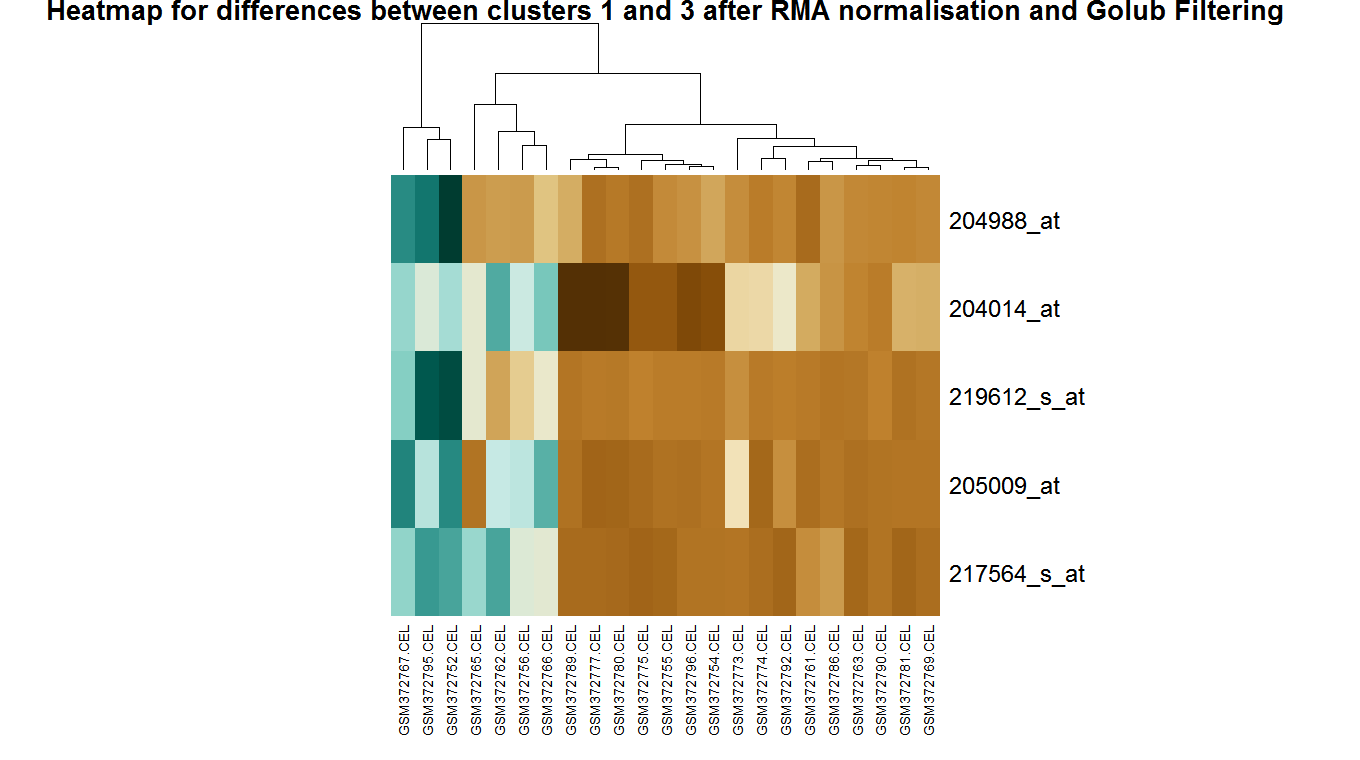

Supplement: Figure S30 — Heatmap for the differentially expressed genes between clusters 1 and 3 after Normalisation with RMA and Golub filtering for 300 probes (TIFF) [file pone.0050253.s030.tiff]

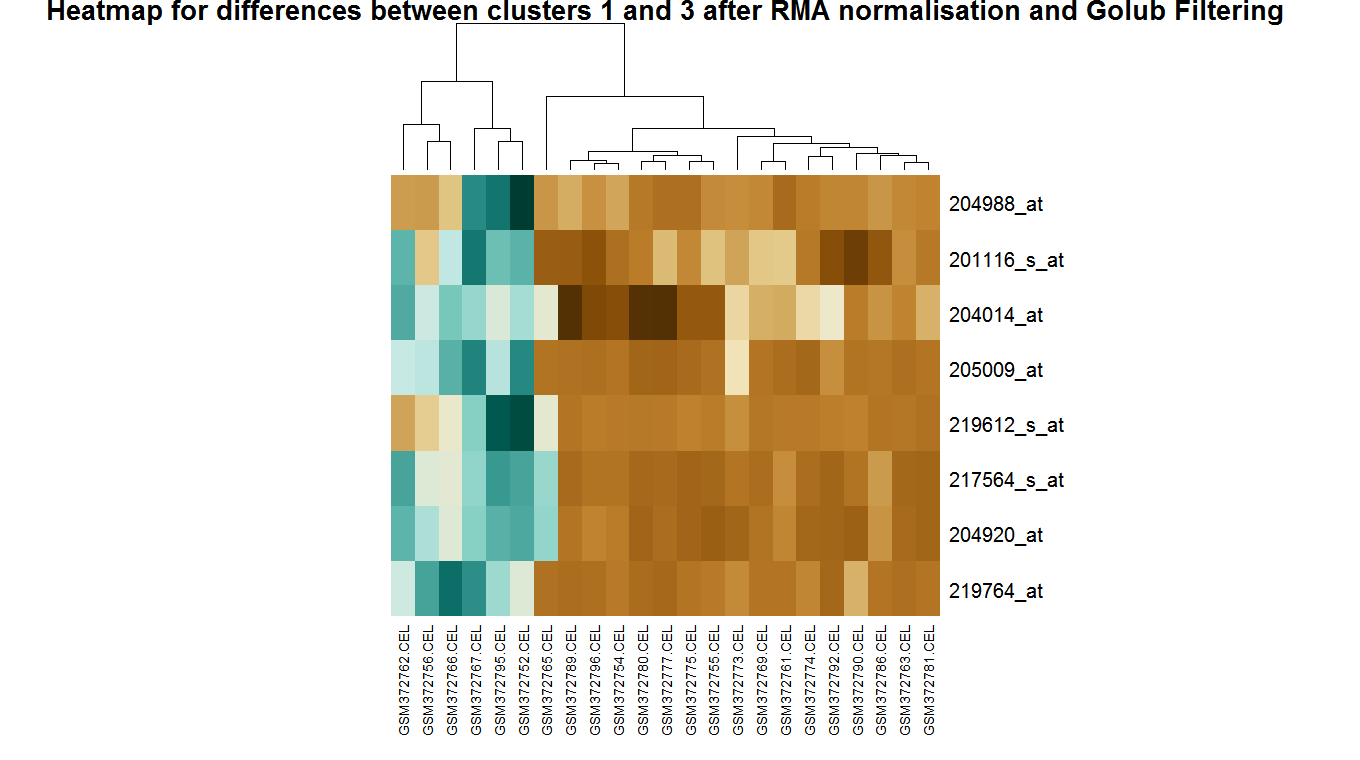

Supplement: Figure S31 — Heatmap for the differentially expressed genes between clusters 1 and 3 after Normalisation with RMA and Golub filtering for 1000 probes (TIFF) [file pone.0050253.s031.tiff]

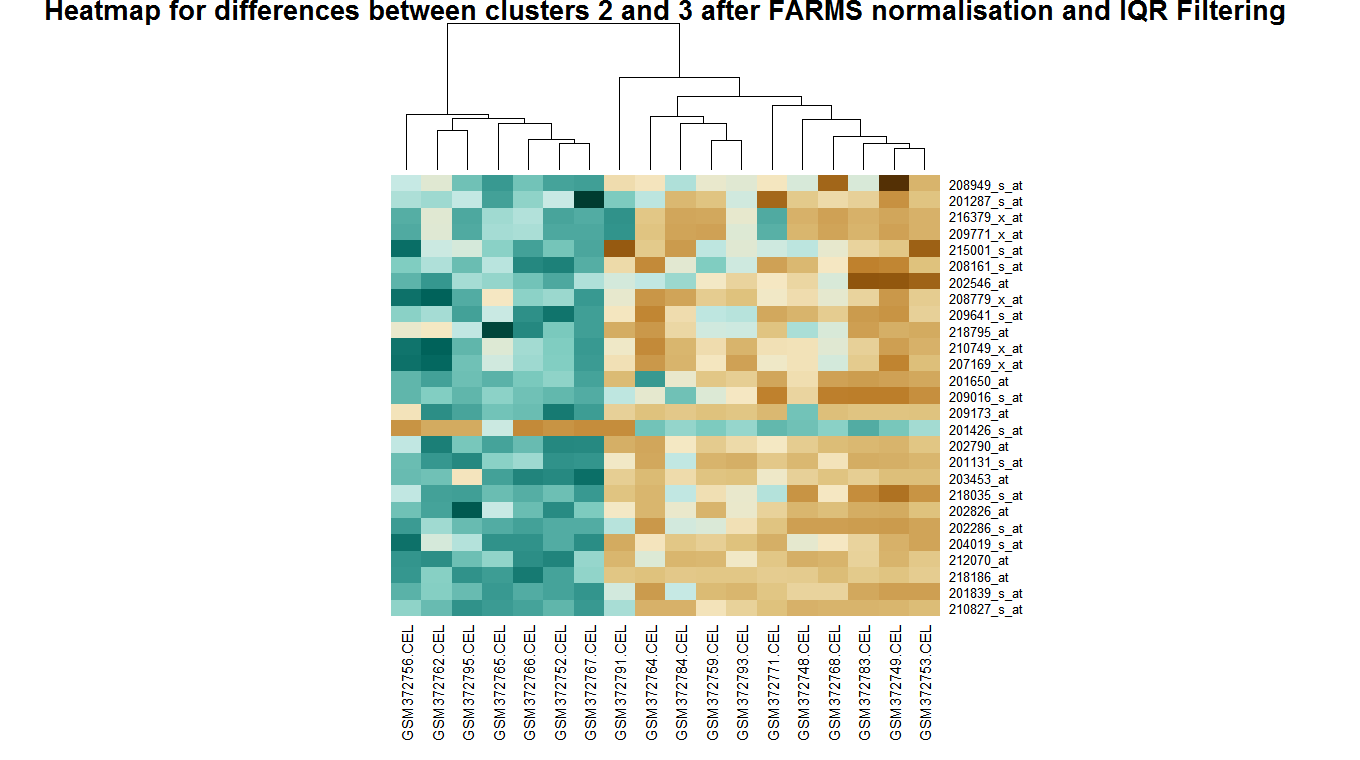

Supplement: Figure S32 — Heatmap for the differentially expressed genes between clusters 2 and 3 after Normalisation with FARMS and IQR filtering for 300 probes. (TIFF) [file pone.0050253.s032.tiff]

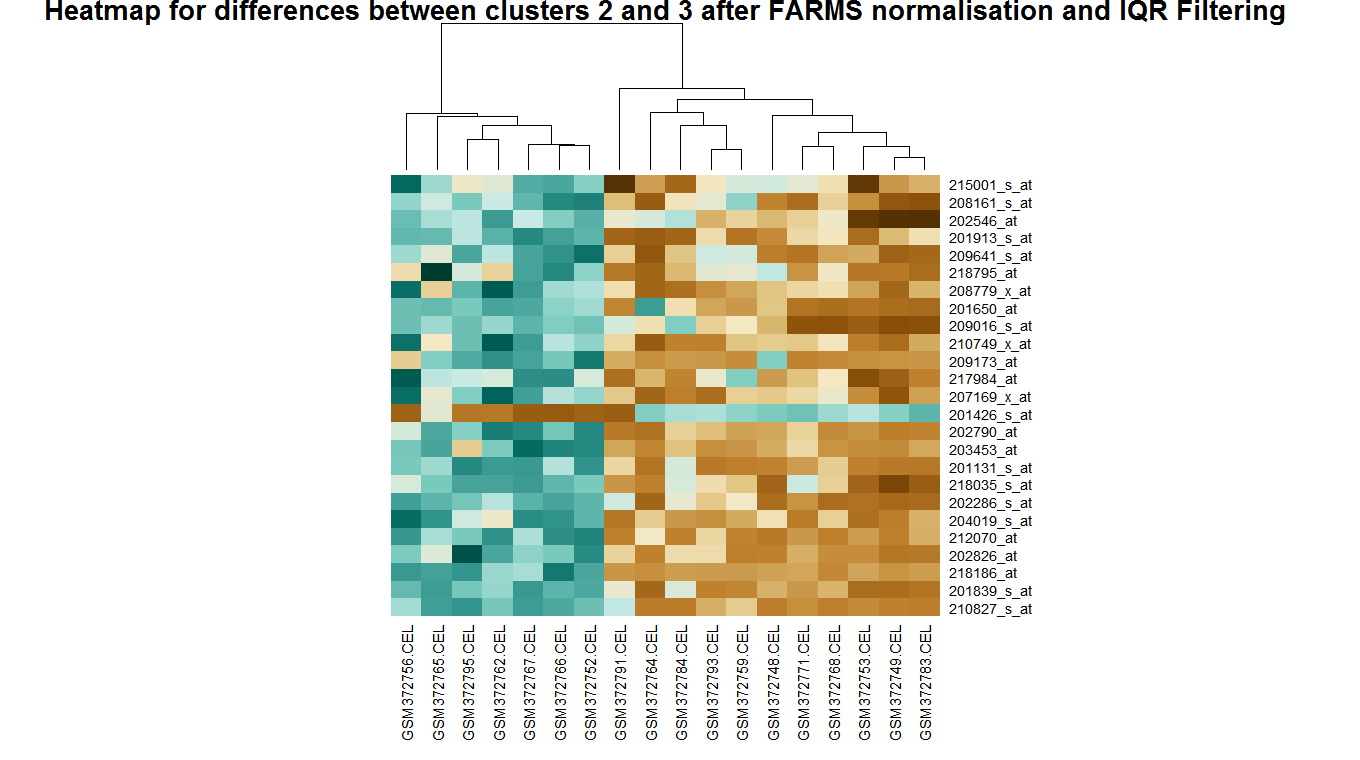

Supplement: Figure S33 — Heatmap for the differentially expressed genes between clusters 2 and 3 after Normalisation with FARMS and IQR filtering for 1000 probes. (TIFF) [file pone.0050253.s033.tiff]

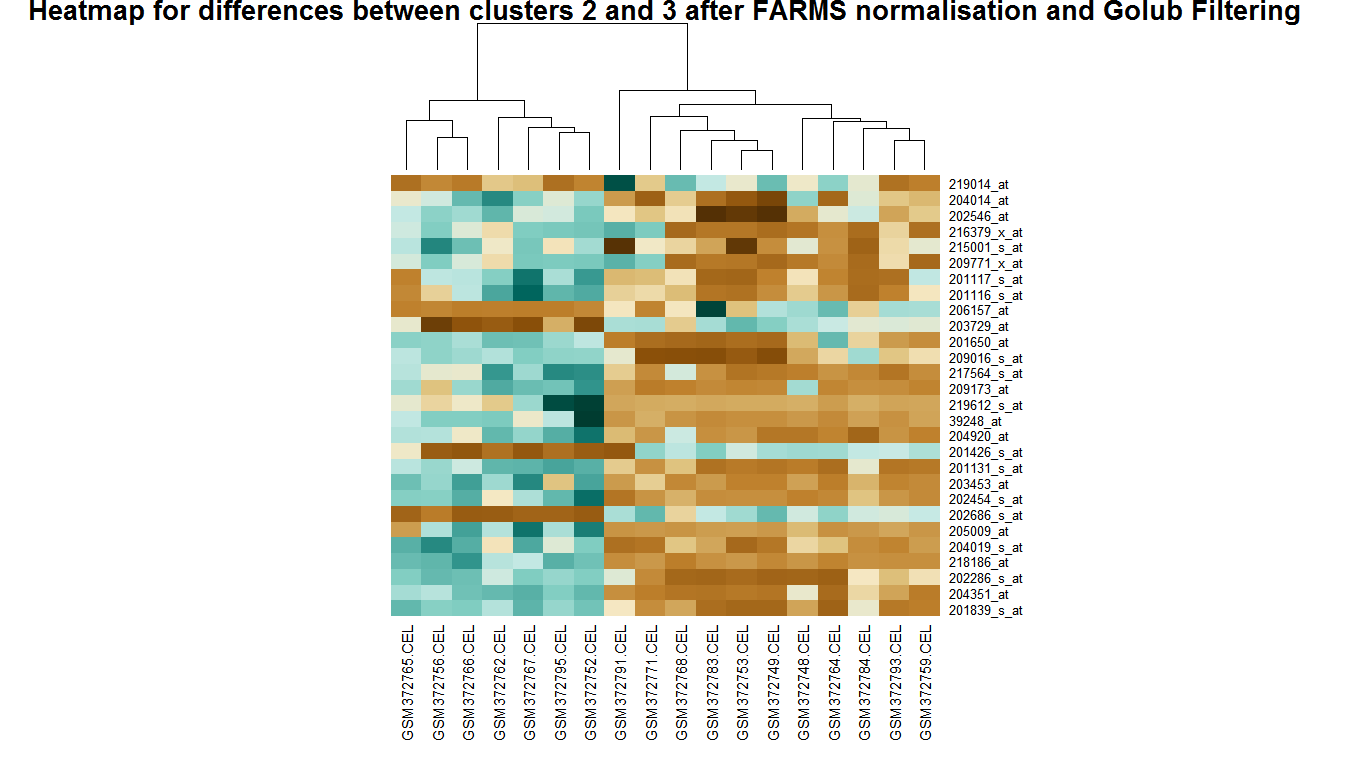

Supplement: Figure S34 — Heatmap for the differentially expressed genes between clusters 2 and 3 after Normalisation with FARMS and Golub filtering for 300 probes. (TIFF) [file pone.0050253.s034.tiff]

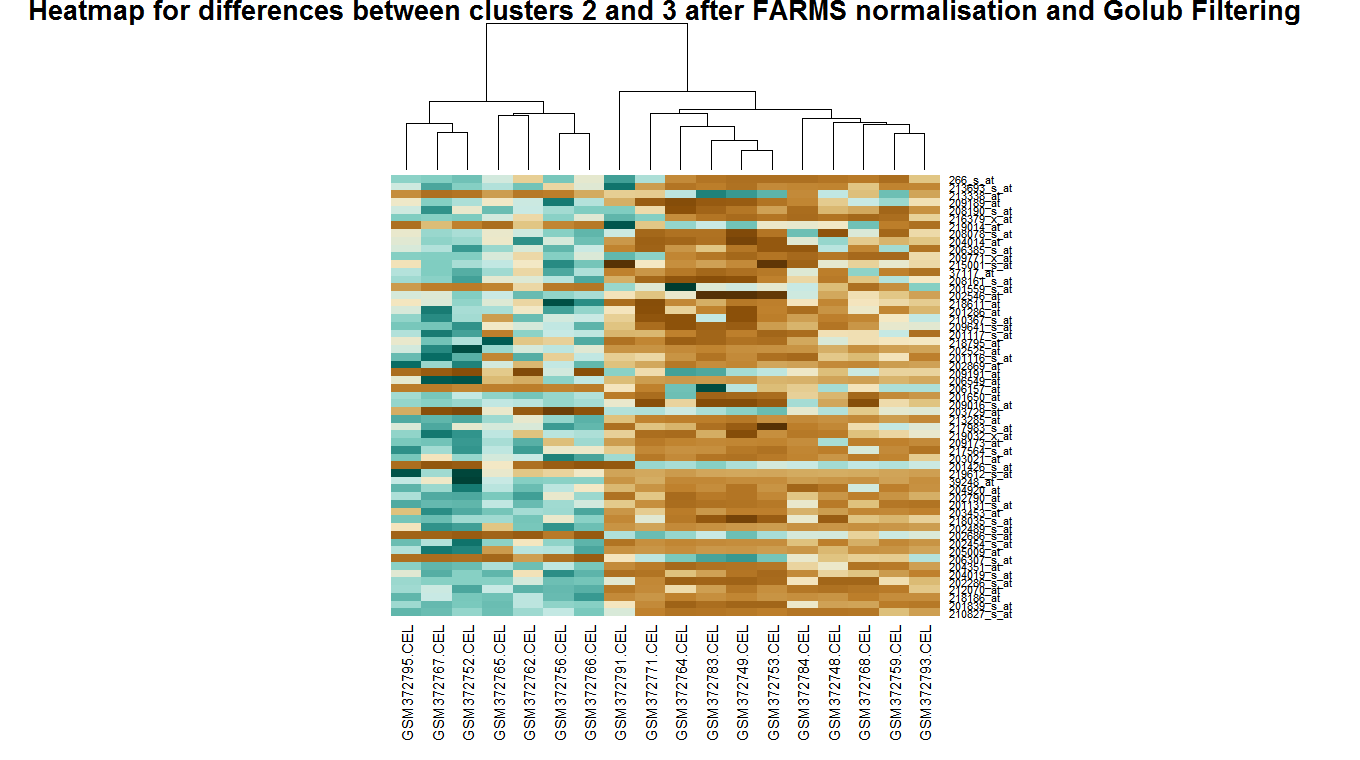

Supplement: Figure S35 — Heatmap for the differentially expressed genes between clusters 2 and 3 after Normalisation with FARMS and Golub filtering for 1000 probes. (TIFF) [file pone.0050253.s035.tiff]

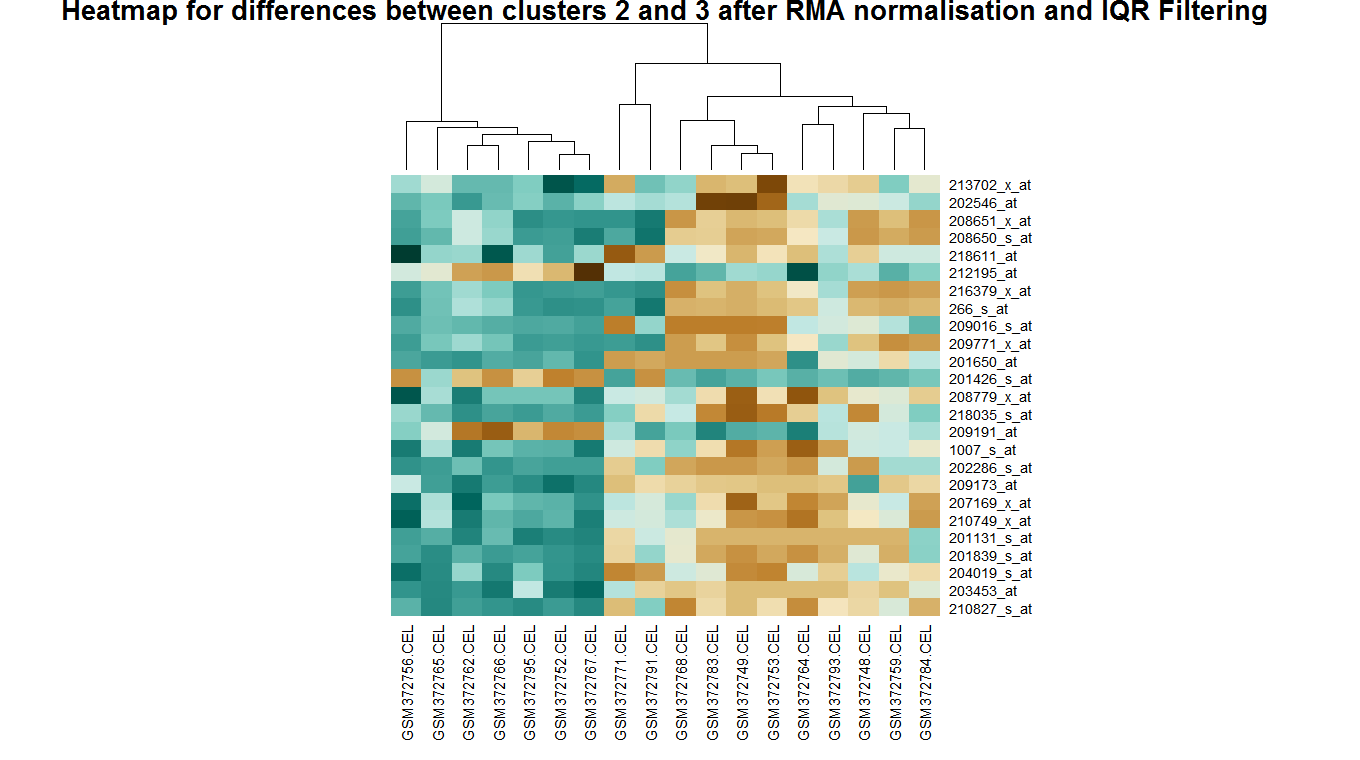

Supplement: Figure S36 — Heatmap for the differentially expressed genes between clusters 2 and 3 after Normalisation with GCRMA and IQR filtering for 300 probes. (TIFF) [file pone.0050253.s036.tiff]

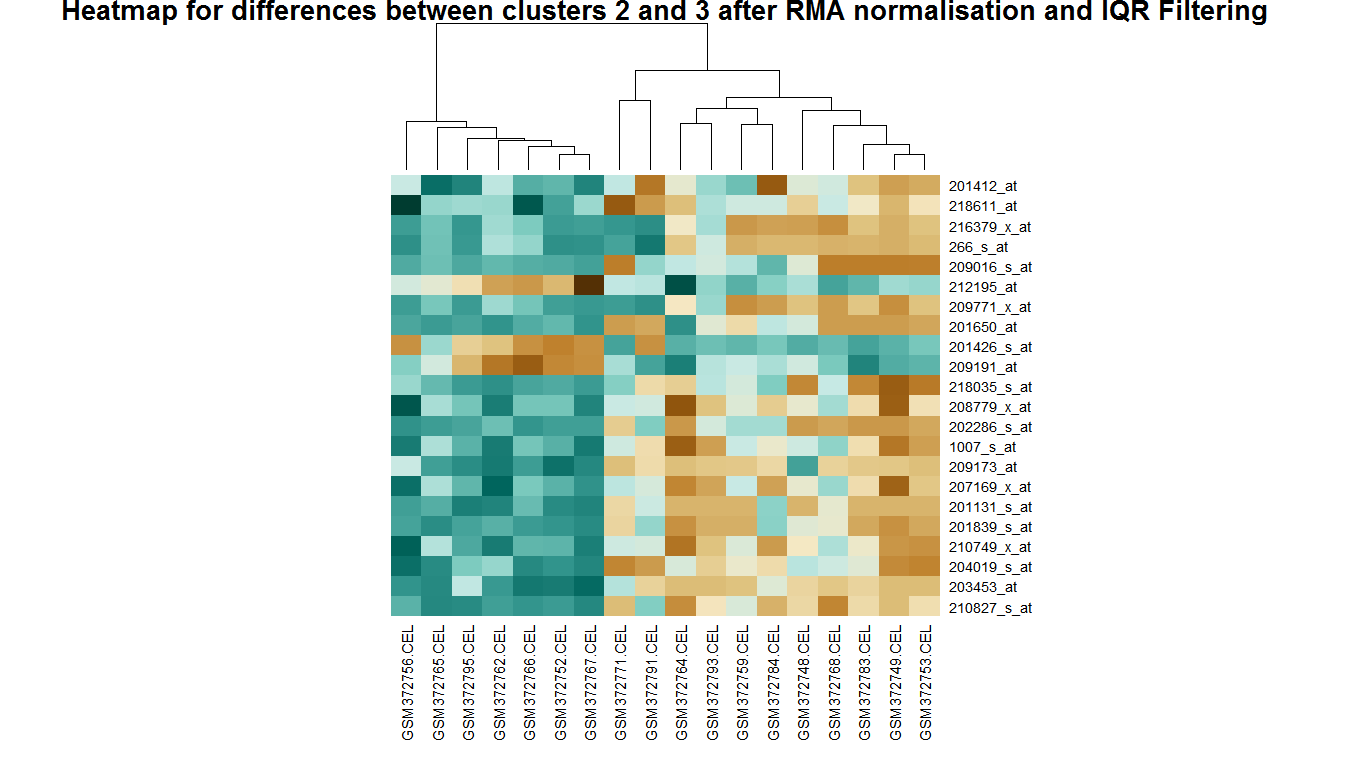

Supplement: Figure S37 — Heatmap for the differentially expressed genes between clusters 2 and 3 after Normalisation with GCRMA and IQR filtering for 1000 probes. (TIFF) [file pone.0050253.s037.tiff]

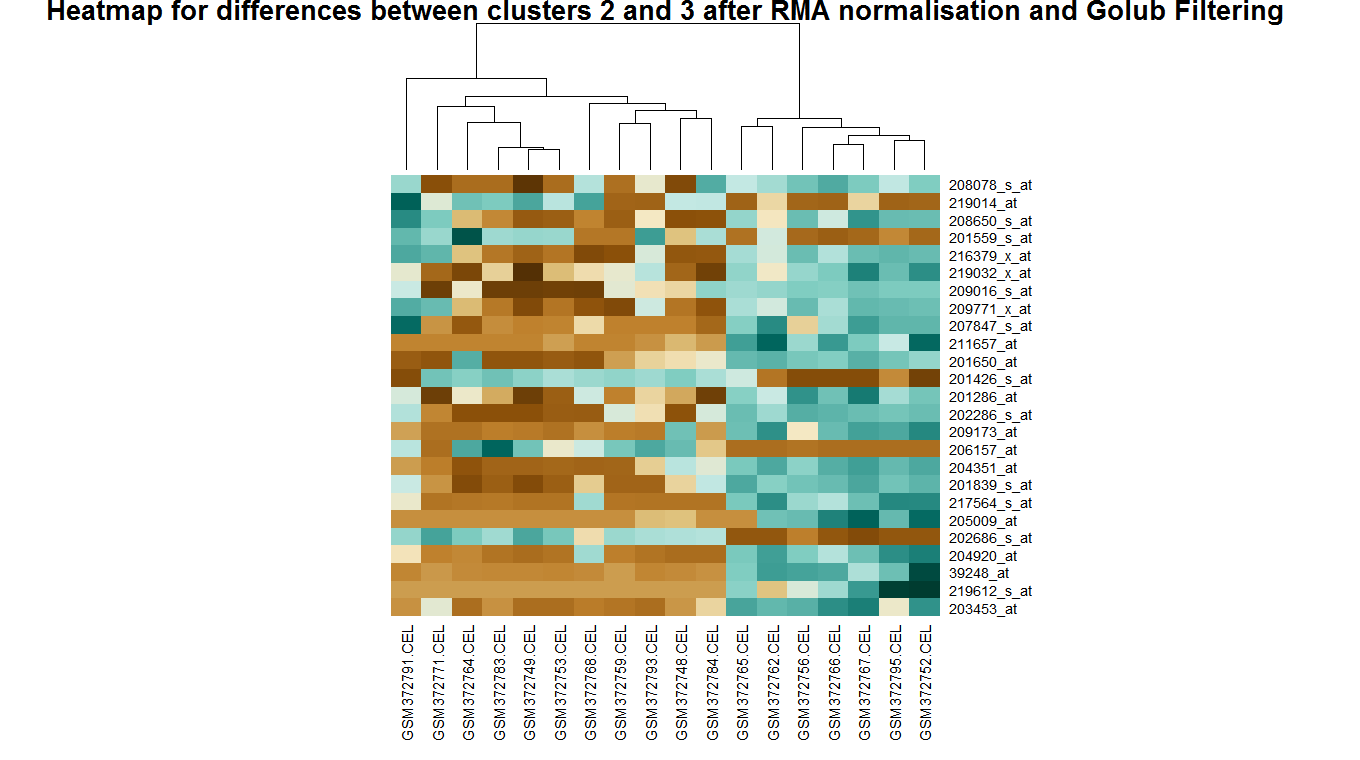

Supplement: Figure S38 — Heatmap for the differentially expressed genes between clusters 2 and 3 after Normalisation with GCRMA and Golub filtering for 300 probes. (TIFF) [file pone.0050253.s038.tiff]

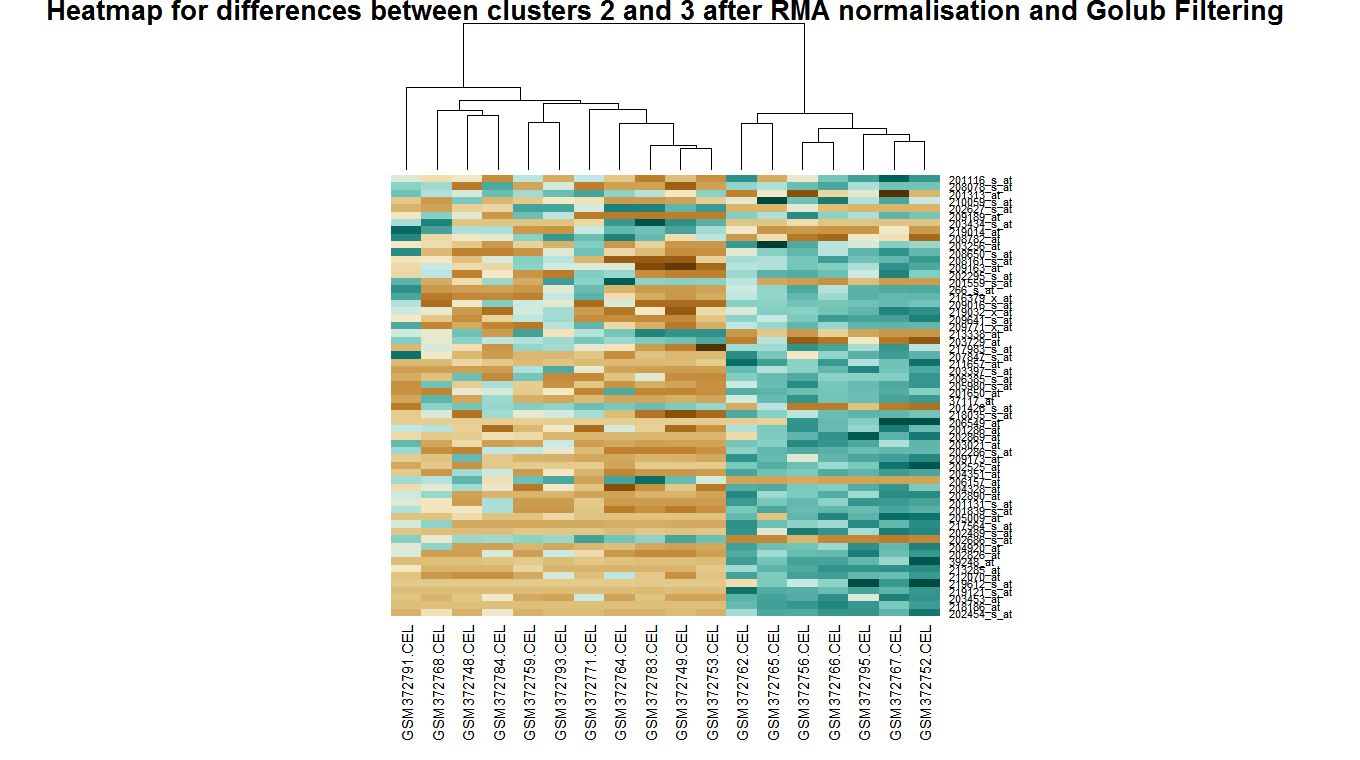

Supplement: Figure S39 — Heatmap for the differentially expressed genes between clusters 2 and 3 after Normalisation with GCRMA and Golub filtering for 1000 probes. (TIFF) [file pone.0050253.s039.tiff]

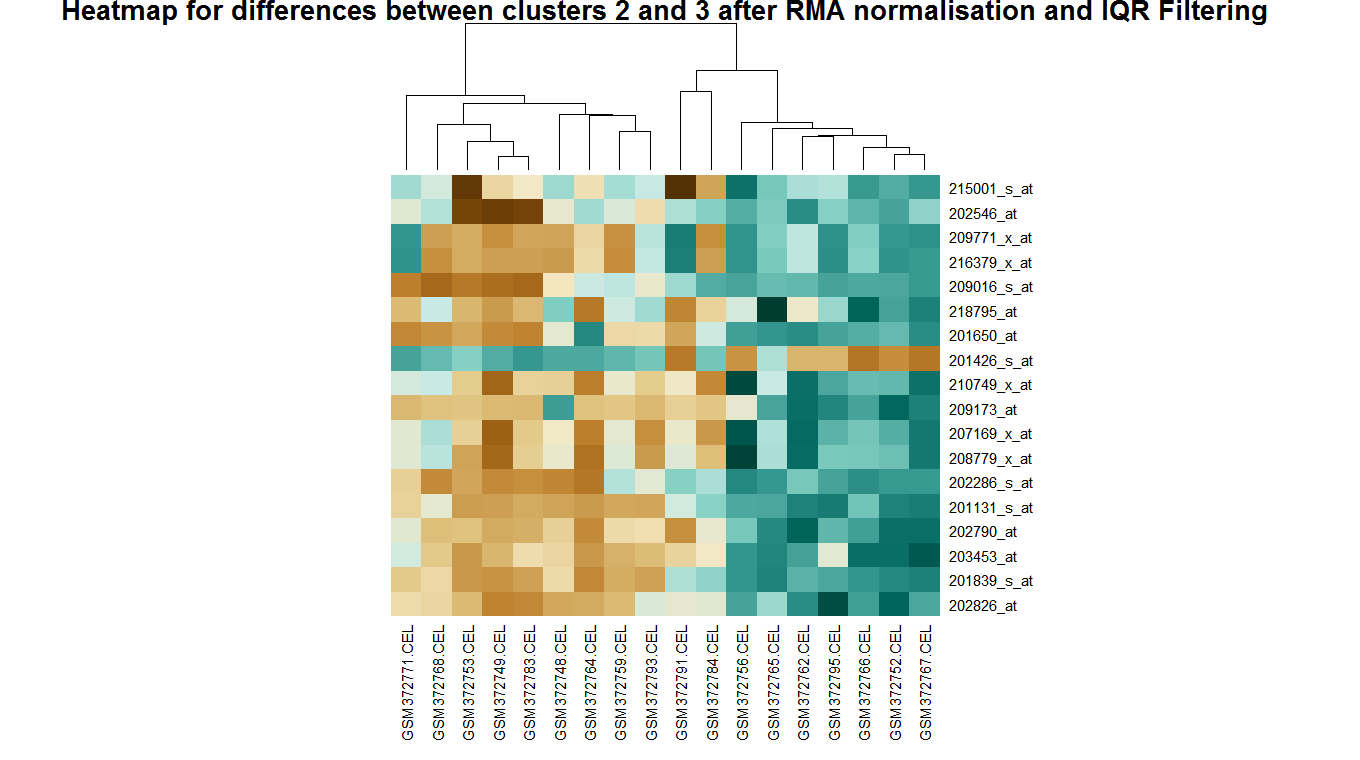

Supplement: Figure S40 — Heatmap for the differentially expressed genes between clusters 2 and 3 after Normalisation with RMA and IQR filtering for 300 probes. (TIFF) [file pone.0050253.s040.tiff]

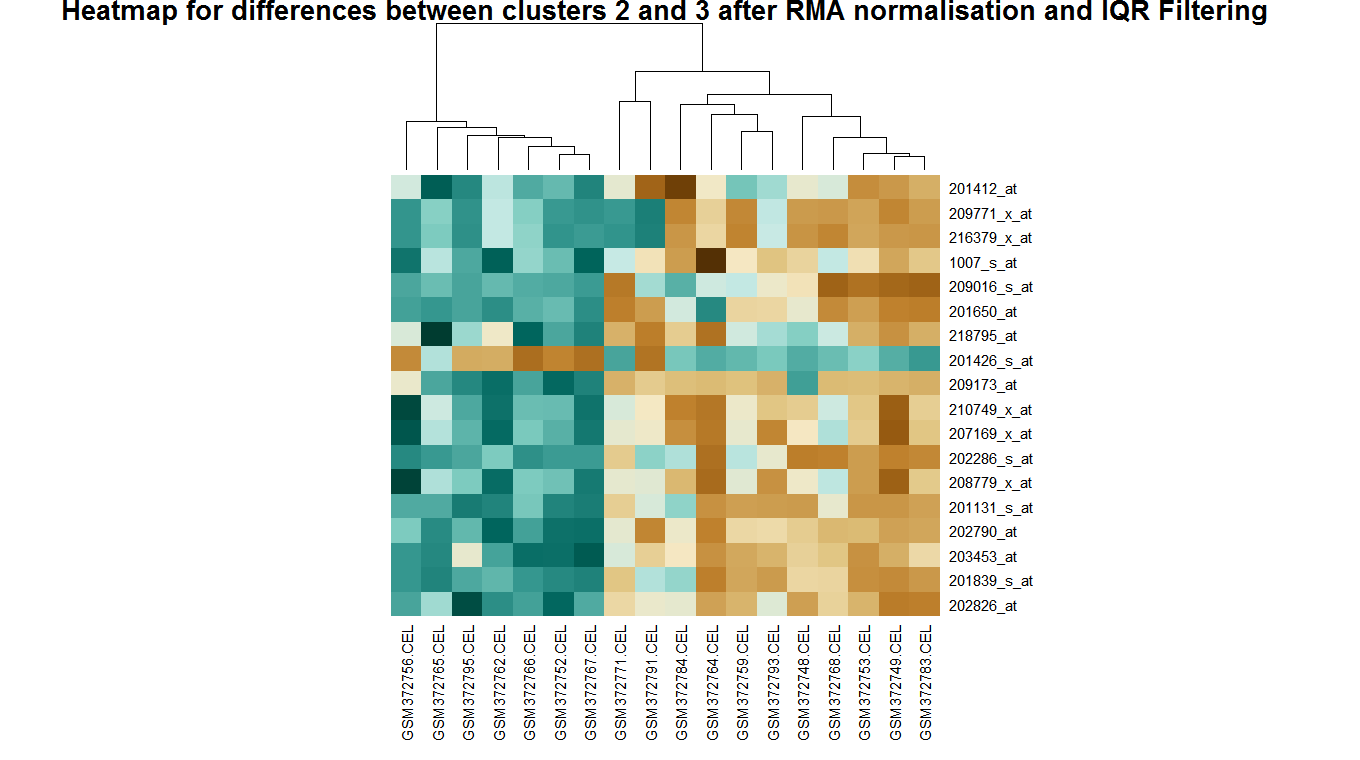

Supplement: Figure S41 — Heatmap for the differentially expressed genes between clusters 2 and 3 after Normalisation with RMA and IQR filtering for 1000 probes. (TIFF) [file pone.0050253.s041.tiff]

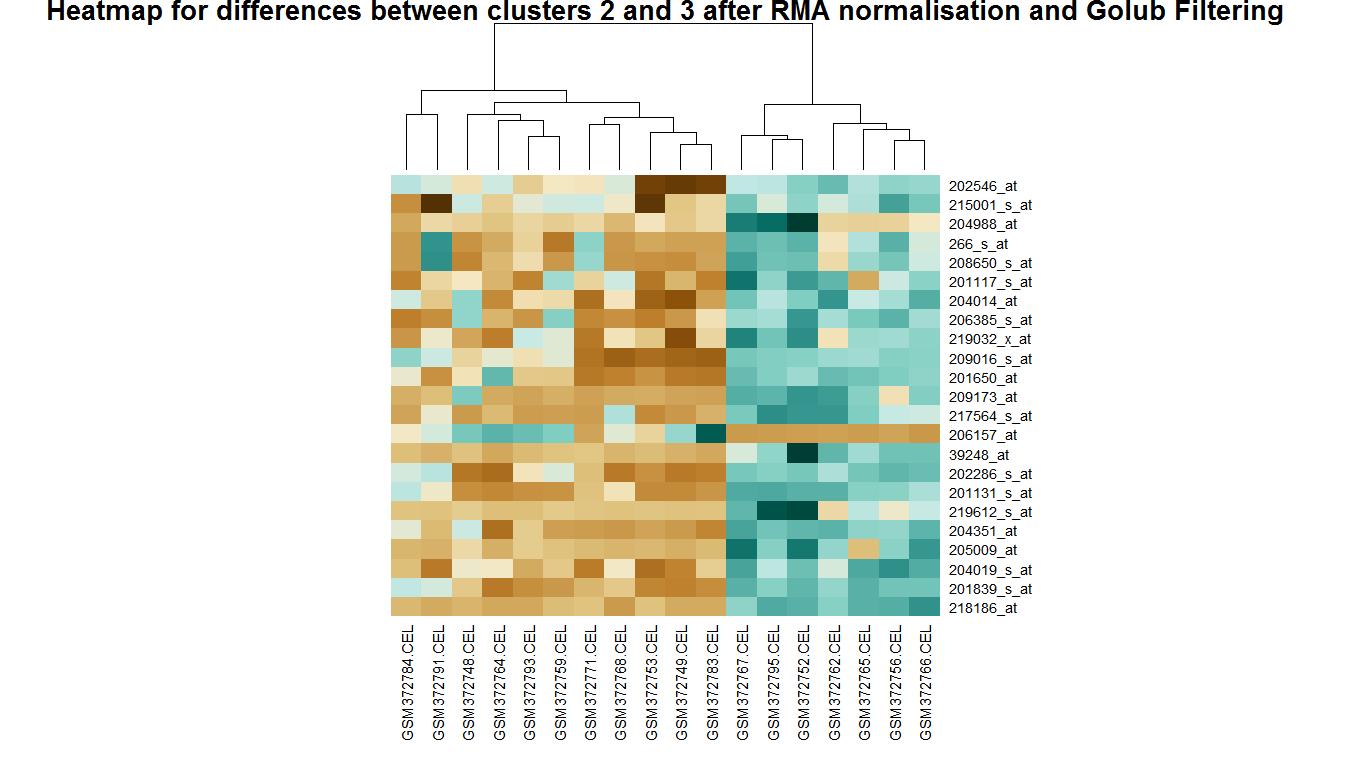

Supplement: Figure S42 — Heatmap for the differentially expressed genes between clusters 2 and 3 after Normalisation with RMA and Golub filtering for 300 probes. (TIFF) [file pone.0050253.s042.tiff]

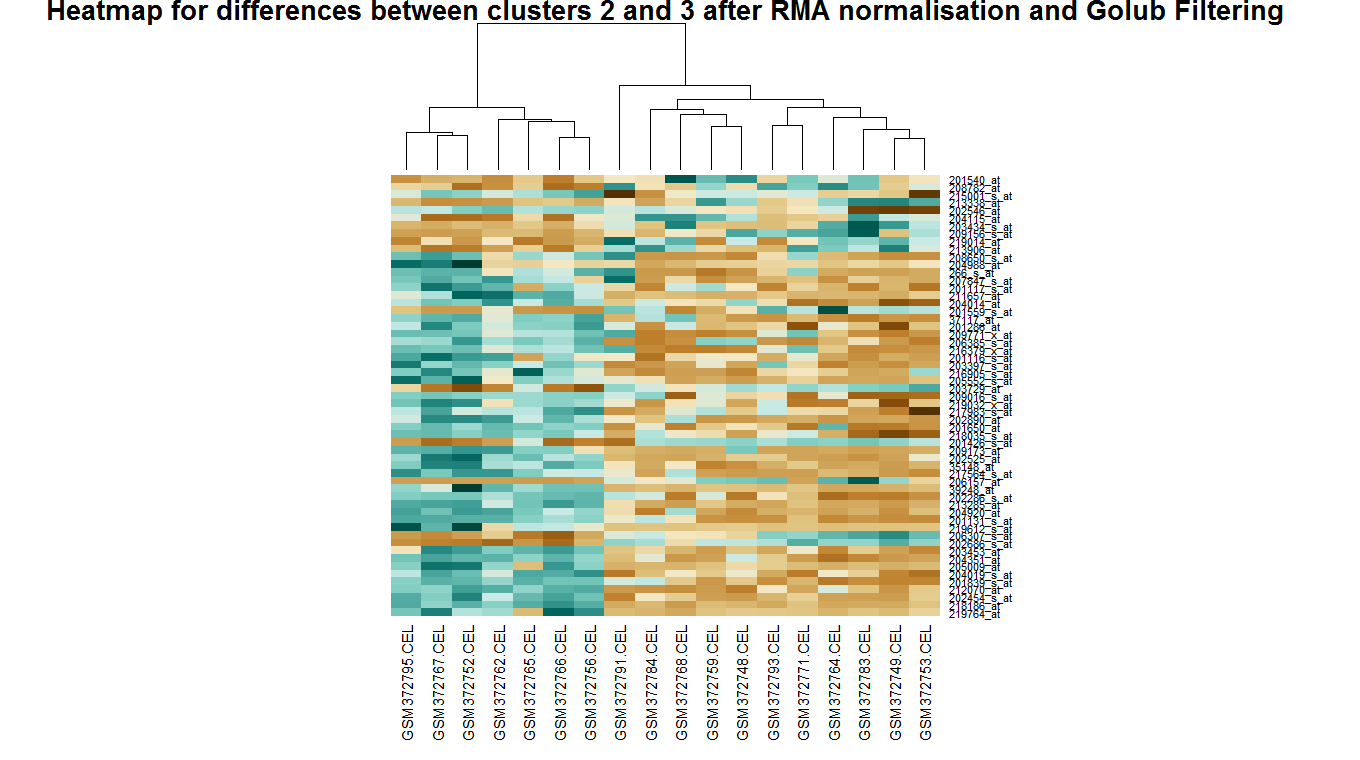

Supplement: Figure S43 — Heatmap for the differentially expressed genes between clusters 2 and 3 after Normalisation with RMA and Golub filtering for 1000 probes. (TIFF) [file pone.0050253.s043.tiff]
